# ALD1 accumulation in Arabidopsis epidermal plastids confers local and non-autonomous disease resistance

**DOI:** 10.1093/jxb/eraa609

**Published:** 2021-01-19

**Authors:** Shang-Chuan Jiang, Nancy L Engle, Zeeshan Zahoor Banday, Nicolás M Cecchini, Ho Won Jung, Timothy J Tschaplinski, Jean T Greenberg

**Affiliations:** 1 Department of Molecular Genetics and Cell Biology, The University of Chicago, Chicago, IL, USA; 2 Oak Ridge National Laboratory, Oak Ridge, TN, USA; 3 University of Edinburgh, UK

**Keywords:** ALD1, Arabidopsis, dexamethasone-inducible expression, epidermal plastid, plant immunity, *Pseudomonas syringae*, systemic acquired resistance

## Abstract

The Arabidopsis plastid-localized ALD1 protein acts in the lysine catabolic pathway that produces infection-induced pipecolic acid (Pip), Pip derivatives, and basal non-Pip metabolite(s). ALD1 is indispensable for disease resistance associated with *Pseudomonas syringae* infections of naïve plants as well as those previously immunized by a local infection, a phenomenon called systemic acquired resistance (SAR). *Pseudomonas syringae* is known to associate with mesophyll as well as epidermal cells. To probe the importance of epidermal cells in conferring bacterial disease resistance, we studied plants in which ALD1 was only detectable in the epidermal cells of specific leaves. Local disease resistance and many features of SAR were restored when ALD1 preferentially accumulated in the epidermal plastids at immunization sites. Interestingly, SAR restoration occurred without appreciable accumulation of Pip or known Pip derivatives in secondary distal leaves. Our findings establish that ALD1 has a non-autonomous effect on pathogen growth and defense activation. We propose that ALD1 is sufficient in the epidermis of the immunized leaves to activate SAR, but basal ALD1 and possibly a non-Pip metabolite(s) are also needed at all infection sites to fully suppress bacterial growth. Thus, epidermal plastids that contain ALD1 play a key role in local and whole-plant immune signaling.

## Teaser

The transient accumulation of ALD1 in epidermal plastids at local immunization sites activates both local disease resistance and systemic acquired resistance in the distal leaves.

## Introduction

Plant disease is a threat to global food security and agricultural sustainability. In either natural or agricultural ecosystems, plants are potential hosts for a broad variety of pathogens (Staal and Dixelius, 2007; Anderson *et al.*, 2010; Andersen *et al.*, 2018; van der Burgh and Joosten, 2019). After pathogens overcome physical barriers to gain access to plant cells, plant immune receptors can recognize pathogens and trigger a defense response (Fu and Dong, 2013; Muthamilarasan and Prasad, 2013). Activation of plant immunity involves responses to pathogen- or microbial-associated molecular patterns (PAMPs or MAMPs) and secreted pathogen effectors to give PAMP- or effector-triggered immunity (PTI and ETI, respectively). During PTI, transmembrane pattern recognition receptors (PRRs) perceive extracellular PAMPs/MAMPs and induce defense programs, whereas ETI is activated by the R protein immune receptor response to pathogen effectors (Jones and Dangl, 2006). Upon a localized infection, primary infected cells at the immunization site can trigger systemic acquired resistance (SAR) (Durrant and Dong, 2004), a long-distance, long-lasting immune response against a broad spectrum of pathogens in the distal tissues, which are also called secondary or distal infection sites. Both effectors and PAMPs/MAMPs can trigger SAR in Arabidopsis (Durrant and Dong, 2004; Mishina and Zeier, 2007; Fu and Dong, 2013). SAR is associated with a primed state that promotes a stronger and/or faster defense response upon a secondary infection in distal tissues (Ryals *et al.*, 1996; Jung *et al.*, 2009).

Plastids are key defense organelles that control the biosynthesis of defense-related molecules, including several plant hormones and secondary messengers (Serrano *et al.*, 2016). Among them, the plant defense hormone salicylic acid (SA) is critical for SAR (Gaffney *et al.*, 1993; Ryals *et al.*, 1996; Rekhter *et al.*, 2019; Zhang and Li, 2019). In Arabidopsis, ISOCHORISMATE SYNTHASE1 (ICS1) is a major chloroplast-localized enzyme in the SA synthesis pathway (Strawn *et al.*, 2007; Rekhter *et al.*, 2019; Torrens-Spence *et al.*, 2019). In plant leaves, the morphology and number of chloroplasts in mesophyll cells are significantly different from those of the chloroplasts in epidermal cells (Barton *et al.*, 2018; Beltrán *et al.*, 2018). However, as the majority of studies focus on chloroplasts found in the leaf mesophyll tissue, the function of epidermal cell chloroplasts in defense against bacterial pathogens is largely unclear (Barton *et al.*, 2018; Beltrán *et al.*, 2018). Therefore, elucidating whether the signals produced from chloroplasts of epidermal cells play a significant role in local and/or whole-plant disease resistance will help discern the potential of epidermal cells to control bacterial diseases.

An important chloroplast-localized defense protein is the aminotransferase AGD2-LIKE DEFENSE RESPONSE PROTEIN 1 (ALD1), which is essential for local disease resistance and SAR (Song *et al.*, 2004*a*, *b*; Cecchini *et al.*, 2015*a*). In uninfected distal leaves of *ald1* mutant plants, SAR-associated events such as SA and *PATHOGENESIS RELATED GENE1* (*PR1*) transcript accumulation prior to secondary infection do not occur (Song *et al.*, 2004*b*). ALD1 is involved in the pathogen-inducible route of l-Lys catabolism resulting in the biosynthesis of the two non-protein amino acid-derived defense signals l-pipecolic acid (Pip) and *N*-hydroxypipecolic acid (N-OH-Pip; NHP). After ALD1 transaminates l-Lys, the reductase SAR-deficient 4 (SARD4) subsequently reduces the intermediate to Pip, which is further hydroxylated by FLAVIN-DEPENDENT MONOXYGENASE 1 (FMO1) to NHP (Návarová *et al.*, 2012; Ding *et al.*, 2016; Hartmann *et al.*, 2017, 2018; Chen *et al.*, 2018; Hartmann and Zeier, 2019). Pip and/or its derivatives have been proposed to regulate SAR and the priming of associated defenses mainly through an SA-dependent signaling pathway (Bernsdorff *et al.*, 2016; Hartmann *et al.*, 2018).

In Arabidopsis, watering the root system of soil-grown plants with exogenous Pip allows leaves to accumulate levels of Pip similar to distal leaves during SAR induced by *Pseudomonas syringae* (Návarová *et al.*, 2012). Such exogenous Pip application is sufficient to enhance plant resistance to *P. syringae*, induce defense priming, and up-regulate a set of immune-regulatory and defense-related genes in the plant (Návarová *et al.*, 2012; Bernsdorff *et al.*, 2016; Hartmann *et al.*, 2018). Additionally, Pip can be detected in vascular exudates after a local infection (Návarová *et al.*, 2012; Wang *et al.*, 2018), and the transport of ^14^C-labeled Pip can be detected in distal leaves after a local application (Wang *et al.*, 2018). Nevertheless, only infusion with NHP, not Pip, of lower leaves causes the accumulation of defense-related gene transcripts in local or distal leaves, including *ALD1*, *SARD4*, *FMO1*, *ICS1*, and *PR1* (Chen *et al.*, 2018). Furthermore, Chen *et al.* (2018) reported that there was no detectable endogenous free NHP at local infection sites of wild-type (WT) seedlings or adult plants. Thus, it is still unclear under natural conditions whether/how Pip and NHP might directly contribute to the long-distance communication between the immunization site and secondary infection site. Additionally, ALD1 also regulates another non-Pip metabolite(s) during basal conditions that is needed for the maintenance of the correct levels of some PRRs associated with PTI and is necessary for a normal local defense response (Cecchini *et al.*, 2015*a*). Therefore, a spatial- and tissue-specific study of ALD1 activation is required to fully understand ALD1’s site of action in defense.

Here, we took advantage of *ald1* mutant plants in which ALD1 is provided from a transgene only detected in epidermal cells to test whether ALD1 in epidermal plastids is sufficient to explain ALD1’s roles in defense. In such chimeric plants, both local disease resistance and the response gain of SAR were restored even when ALD1 only accumulated at the immunization site. This suggests a critical role for epidermal plastids and the plastid protein ALD1 in both local and systemic plant immune signaling to suppress the bacterial pathogen *P. syringae*.

## Materials and methods

### Arabidopsis plants and growth conditions

All plants used in this study were in the *Arabidopsis thaliana* Columbia-0 (Col-0) background. Col-0 is used as the WT. The *ald1-T2* (SALK_007673) mutant was described previously (Song *et al.*, 2004*a*, *b*). The transgenic marker line pt-gk from the ABRC (CS16266) was used as a control for general chloroplast localization of green fluorescent protein (GFP). Plants were grown under 12 h light (08.00 h to 20.00 h) and 12 h dark conditions at 20 °C, as described (Jung *et al.*, 2009; Cecchini *et al.*, 2015*a*, *b*).

*pDEX::ALD1:GFP* (hereafter called *pDEX::ALD1*) transgenic plants were generated as described (Cecchini *et al.*, 2015*a*), with GFP fused to the C-terminus of ALD1 and controlled by a dexamethasone (DEX)-inducible promoter in the pBAV150 vector in the *ald1-T2* mutant background. In order to select *pDEX::ALD1* transgenic lines, the seeds (*n*≥100 per line) of 10 independent lines in the T_2_ generation were directly planted on half-strength Murashige and Skoog (MS) medium (Sigma-Aldrich, Saint Louis, MO, USA) containing BASTA 10 μg ml^–1^ (Sigma-Aldrich). The χ ^2^ test (Snedecor and Cochran, 1989) was used to test the phenotypic ratio of a single insertion in transgenic plants. Two homozygous lines, #6 and #10, in the T_3_ generation were used for experiments.

### Dexamethasone treatments

To induce ALD1:GFP expression only in specific leaves, selected leaves were gently painted (1/4'' Angler Shader paintbrush, Princeton Art and Brush Co.) with DEX (Sigma-Aldrich) solution (typically 30 μM, except where indicated) plus 0.04% Tween-20. The same amount of the solvent ethanol as the DEX stock solution was used for mock treatments. For subcellular localization of DEX-inducible ALD1:GFP, leaves of transgenic plants were infiltrated or soaked by DEX water solution, or sprayed with DEX solution plus 0.04% Tween-20 at the indicated concentrations.

### Accumulation and subcellular localization of GFP signals

GFP and ALD1:GFP fusion proteins were visualized by confocal microscopy as described (Cecchini *et al.*, 2015*a*, *b*). Zeiss LSM710 and LSM800 laser scanning confocal microscopes (Carl Zeiss Microscopy GmbH, Germany) were used to visualize GFP fluorescence and chlorophyll autofluorescence. Images for GFP and plant autofluorescence were acquired for the same field using a sequential acquisition mode. Images, Z-series sections, and 3D videos were processed using Zen 2.3 Blue Edition (Carl Zeiss Microscopy GmbH) and Adobe Photoshop software. For *Z*-series acquisition for Ortho view (maximum intensity projections of *Z*-series images) and 3D View (displays images three-dimensionally for movie export), images were taken at above 512×512 pixels scanning resolution. The plant tissues were first treated with perfluorodecalin (Strem Chemicals, Inc., Newburyport, MA, USA) to enhance the *in vivo* confocal microscopy resolution (Littlejohn *et al.*, 2010). About 60 slices (60–65 μm) were taken along the *Z*-axis. For observation of the epidermal and mesophyll tissue, the epidermal strips were peeled with tweezers from the abaxial surface of the leaf, and immediately transferred into a water drop on a microscope slide. A clean, soft brush can be used to unfold the epidermis tissue. The leaf tissue from the corresponding peeled region was observed to detect the signal in the mesophyll layer.

### Pathogen infections and establishment of systemic acquired resistance

SAR was induced as described previously (Jung *et al.*, 2009; Cecchini *et al.*, 2015*b*). Lower leaves (the third to fifth leaves) of 26-day-old plant grown were inoculated with the avirulent derivative of *Pseudomonas cannabina* pv. *alisalensis* (Bull *et al.*, 2010), formerly named *Pseudomonas syringae* pv. *maculicola* strain ES4326, carrying *avrRpt2* (*Pma*DG6) or *avrRpm1* (*Pma*DG34), at OD_600_=0.01. Two days later, the primary inoculated leaves were removed before the secondary inoculation. Upper leaves (the sixth to eighth leaves) were inoculated with a virulent *P. syringae* pv. *maculicola* (*P. cannabina* pv. *alisalensis*) strain ES4326 carrying an empty vector (*Pma*DG3, OD_600_=0.0001–0.0002). Bacterial growth was determined from different infected plants 3 d after inoculation as described previously (Greenberg *et al.*, 2000). Colony-forming unit (CFU) values were converted to log_10_ CFU values.

### RNA preparation, cDNA synthesis, and qPCR analyses

Total RNA preparations were carried out with Trizol reagent (Thermo Scientific, Rockford, lL, USA) or the RNeasy Plant Mini Kit (QIAGEN, Hilden, Germany) according to the manufacturer’s instructions, supplemented with DNA digestion (RNase-free DNase I, New England Biolabs, Ipswich, MA, USA; RNase-Free DNase Set, QIAGEN). cDNA synthesis was conducted with Reverse Transcriptase SuperScript III and oligo(dT)_20_ primer (Thermo Scientific) according to the manufacturer’s instructions.

Quantitative real-time PCR (qPCR) was performed as described (Mei *et al.*, 2014; Jiang *et al.*, 2015). The cDNA was amplified using SYBR Premix Ex Taq (Takara Bio USA, Inc., Mountain View, CA, USA) in a 10 μl volume, according to the instructions provided for the Bio-Rad Real-Time System CFX96TM C1000 thermal cycler (Bio-Rad, Hercules, CA, USA). Data were collected and analyzed using the Bio-Rad CFX Manager 3.1 software (Bio-Rad). Amplification of *ACTIN2* (*ACTIN*) and *EF1A* genes was used as an internal control. For gene primer sequence, see Supplementary Table S1.

### Petiole exudate collection

The petiole exudate collection method containing EDTA in the final extract was conducted as described previously (Jung *et al.*, 2009; Cecchini *et al.*, 2015*a*) with some modifications. *pDEX::ALD1*, WT, and *ald1-T2* plants at ~4 weeks old were sprayed with 30 μM DEX for 24–48 h before or after inoculation with 10 mM MgSO_4_ or *Pma*DG6 (OD_600_=0.01). The third to sixth leaves were then excised and petioles of leaves were surface sterilized in 50% ethanol, and placed in a solution of 1 mM Na_2_-EDTA (pH 8.0) after removing the ethanol. The bases of 12 petioles were recut and stacked so the cut petioles were aligned. Finally, 12 petioles were submerged in 1.4 ml of 1 mM Na_2_-EDTA (pH 8.0) solution supplemented with carbenicillin (50 μg ml^–1^) and streptomycin (50 μg ml^–1^) from 12 h to 72 h after infection.

Tubes were kept in a growth chamber (20 °C, 16 h light and 8 h dark) inside Ziploc bags with wet paper to retain humidity. At the intended collection time, leaves were removed and the exudates were centrifuged three times at 12 000 rpm for 10 min. Supernatants of exudates were immediately frozen in liquid nitrogen and stored at –80 °C until use.

### HPLC analysis of salicylic acid

SA was extracted from 3–4 biological replicates per genotype and analyzed by HPLC as previously described (Zhang *et al.*, 2017). Data were corrected for recovery using samples spiked with *o*-anisic acid as an internal control. Pure SA and *o*-anisic acid (Sigma-Aldrich) were used as standards. SA and *o*-anisic acid content was determined by fluorescence (SA, excitation 301 nm, emission 412 nm; *o*-anisic acid, excitation 301 nm, emission 365 nm) after separation on a C18 reverse-phase HPLC column (ZORBAX SB-C18, Agilent Technologies, TN, USA) with the Agilent Technologies 1200/1100 series LC system. The column was maintained at 25 °C, and methanol:0.5% glacial acetic acid (60:40, v/v) was flowed through at a rate of 1.25 ml min^–1^ for ~20 min.

### GC-MS analysis of metabolites

In this study, we employed GC-MS for the quantification of Pip and other target metabolites in both plant leaf tissue and petiole exudates. For metabolite analyses in the plant tissue, the protocol was as described previously (Tschaplinski *et al.*, 2012, 2014; Abraham *et al.*, 2016). Briefly, 80 mg of ground frozen Arabidopsis leaf tissue was extracted with ethanol (80%) to which sorbitol was added as internal standard. A 1 ml or 1.5 ml aliquot of the extract was dried in a stream of nitrogen and used for derivatization and analysis. For the metabolite analyses in petiole exudates, the protocol was as described previously (Jung *et al.*, 2009; Cecchini *et al.*, 2015*a*). Briefly, a 500 µl aliquot of Arabidopsis petiole exudate to which sorbitol was added as internal standard was dried in a stream of nitrogen and silylated to produce trimethylsilyl-derivatized metabolites that were analyzed by GC-MS with electron impact ionization (70 eV) using an Agilent Technologies Inc. (Santa Clara, CA, USA) 5975C inert XL GC-MS. Metabolites were identified, and scaling factors for quantification were generated from standards of pure Pip (Sigma-Aldrich), NHP (a kind gift from Dr Elizabeth Sattely, Stanford University and Dr Jürgen Zeier, Heinrich Heine University), SA, and camalexin (Sigma-Aldrich).

### Statistical analysis

All statistical analyses were performed using PRISM (GraphPad Software, Inc., La Jolla, CA, USA). One-way or two-way ANOVA or Student’s *t*-test was used to test for significant differences.

### Response gain calculation and propagation of uncertainties

The parameter ‘gain effect’ was calculated in order to quantitatively evaluate the resistance response variation due to effects such as immunizing infection between genotypes after DEX treatments. As raw CFU values were converted to log_10_ CFU values for SAR data, gain effect of SAR due to the primary (1°) immunizing infection was quantified by:

$$\begin{array}{l} R=log_{10}\left(\frac{{\bar{MP}}_{raw}}{{\bar{PP}}_{raw}}\right)=log_{10}{\bar{MP}}_{raw}-log_{10}{\bar{PP}}_{raw}=\bar{MP}-\bar{PP} \end{array}$$ (1)

and propagation of uncertainty in differences is shown below:

$$\begin{array}{l} \delta R=\sqrt{{\left(\delta MP\right)}^{2}+{\left(\delta PP\right)}^{2}} \end{array}$$ (2)

Where $\bar{MP}$ or $\bar{PP}$ are the average of the log_10_ CFU value after treatment combination of 1°-MgSO_4_/2°-*Pma* or 1°-*Pma*/2°-*Pma*, respectively; and δMP or δPP are the SEM (SD/√*n*) calculated from the log_10_ CFU values.

The gain effect of SA in distal leaves due to 1° immunizing infection was quantified by:

$$\begin{array}{l} R=\bar{PX}/\bar{MX}-1 \end{array}$$ (3)

and propagation of uncertainty in quotients is shown as below:

$$\begin{array}{l} \delta R=\left|R\right|\cdot \sqrt{{\left(\frac{\delta MX}{\bar{MX}}\right)}^{2}+{\left(\frac{\delta PX}{\bar{PX}}\right)}^{2}} \end{array}$$ (4)

where $\bar{MX}$ or $\bar{PX}$ are average values of SA after treatment combination of 1°-MgSO_4_/2°-*X* or 1°-*Pma*/2°-*X*, respectively; *X* can be N (no treatment) or P (*Pma*) according to the indicated experiment conditions at each time point, and δMP or δPP are the SEM (SD/√*n*).

If the results are combined from two or more sets of data, *R* will be the average of the different data sets. For example, if combined from data sets of three independent experiments, the average response gain and uncertainty will be

$$\begin{array}{l} {\left(\underset{i=1}{\overset{3}{\sum }}\;R\right)}_{i}/3\pm \frac{1}{3}\cdot \sqrt{\underset{i=1}{\overset{3}{\sum }}\;{\left(\delta R_{i}\right)}^{2}} \end{array}$$ (5)

Then statistical analyses were employed to compare whether the response gain is significantly different from the WT after DEX or mock treatment in local or distal leaves. Propagation of uncertainties was calculated as described above.

## Results

### Dexamethasone-inducible ALD1:GFP preferentially accumulates in epidermal chloroplasts of leaves

**Fig. 1.**
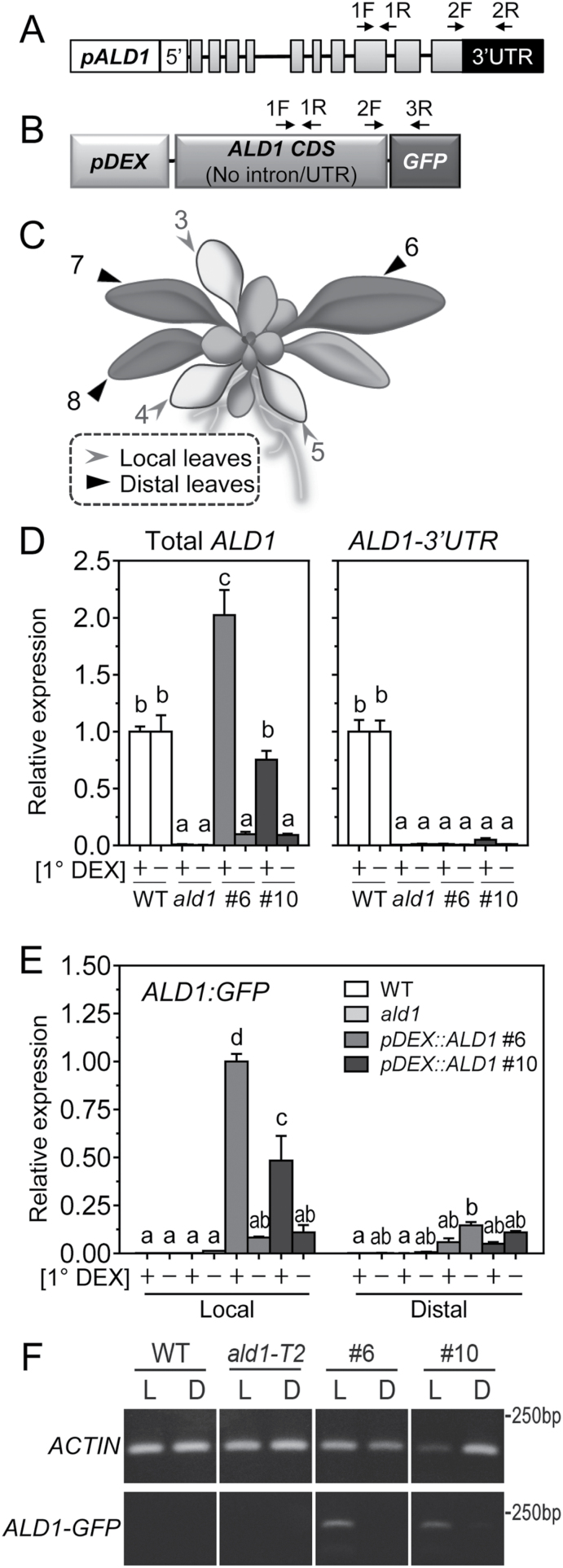
Leaf-specific expression of ALD1 transcripts after treatment with DEX painting. (A) Diagram of the molecular structure of native *ALD1* transcripts. The binding sites of primers used for *ALD1* expression analysis in (D) are indicated: primers 1F and 1R on the *ALD1* CDS is for ‘Total *ALD1*’ revealing both native *ALD1* and transgenic *ALD1:GFP* transcripts in (B); primers 2F and 2R are for ‘*ALD1-3'UTR*’, revealing native *ALD1* transcripts. Gray boxes represent exons, and the black box represents the 3'-untranslated region (UTR). Primer 1R spans the intron. *pALD1*, native promoter of *ALD1* in Arabidopsis. 5', 5'-UTR. (B) Diagram of the pBAV150 plant expression vector containing the DEX-inducible promoter (*pDEX*)-driven GFP-tag-fused *ALD1* sequence in *pDEX::ALD1* transgenic plants. Primer pair 2F and 3R is used for analysis of *ALD1:GFP* in (E), revealing *ALD1:GFP* transgene transcripts. (C) Cartoon showed that the third to fifth leaves (lower leaves) were used as local leaves which will be immunized during the primary infection, and the sixth to eighth leaves (upper leaves) were used as distal leaves which will be challenged in the secondary infection in SAR experiments. Local leaves were painted with 30 μM DEX for ~1 d, while distal leaves were untreated. (D) Relative normalized expression of total *ALD1* and *ALD1-3'UTR* transcripts by qPCR in local leaves of the indicated genotypes: wild type (WT), *ald1-T2* (*ald1*), and *pDEX::ALD1* transgenic lines #6 and #10. The transgenic *pDEX::ALD1* lines #6 and #10 are in the *ald1* mutant background. (E) Relative normalized expression of DEX-inducible transgenic *ALD1:GFP* transcripts by qPCR in treated local leaves and in untreated distal leaves of the indicated genotypes. 1° DEX ‘+’ or ‘–’ in (D) and (E) indicates that local leaves were treated with DEX or mock, respectively. (F) Semi-quantitative RT-PCR of DEX-induced transgenic *ALD1:GFP* levels in DEX-treated local leaves (shown as ‘L’) and in untreated distal leaves (shown as ‘D’) of different genotypes shown in (E). Error bars indicate the SEM from three biological replicates and three technical replicates. Different letters indicate statistically significant differences (*P*<0.05, ANOVA, Fisher’s LSD test). In (D–F), *ACTIN* was used as the internal control.

To study the potential site-specific functions of ALD1, we used *pDEX::ALD1* transgenic lines, which are *ald1-T2* (*ald1* mutant) plants with DEX-inducible ALD1:GFP. We first employed DEX leaf painting to induce and test whether *ALD1:GFP* transcripts were restricted to sites of DEX application. We independently assessed native *ALD1* (Fig. 1A) as well as *ALD1* transcripts driven by the transgene (Fig. 1A, B). We monitored their transcript levels in both local DEX-treated and distal untreated leaves (Fig. 1C). Two independent transgenic lines carrying *pDEX::ALD1* (#6 and #10) were used.

As shown in Fig. 1D, in the local DEX-treated leaves of transgenic lines *pDEX::ALD1* #6 and #10, the total *ALD1* transcripts were significantly induced compared with untreated local leaves, and line #6 showed higher levels. As expected, both *pDEX::ALD1* lines showed no native *ALD1* transcripts (*ALD1-3'UTR*) in DEX- or mock-treated leaves, similar to levels in the control *ald1* plants. Importantly, the *ALD1:GFP* transcript levels in *pDEX::ALD1* lines were only induced at the sites of DEX application (DEX-treated local leaves), but not untreated distal leaves, as assessed by qPCR (Fig. 1E) or semi-quantitative PCR (Fig. 1F). WT plants showed only the expression of native *ALD1* transcript and no transgenic *ALD1* transcript, whereas *ald1* showed no *ALD1* transcript accumulation at all (Fig. 1D–F).

We next analyzed the spatial accumulation of the ALD1:GFP fusion protein by confocal microscopy. Our first analysis of the ALD1:GFP signal localization using confocal microscopy showed fluorescence only in the epidermal cell layer of leaf tissue after DEX infiltration (Supplementary Fig. S1).

**Fig. 2.**
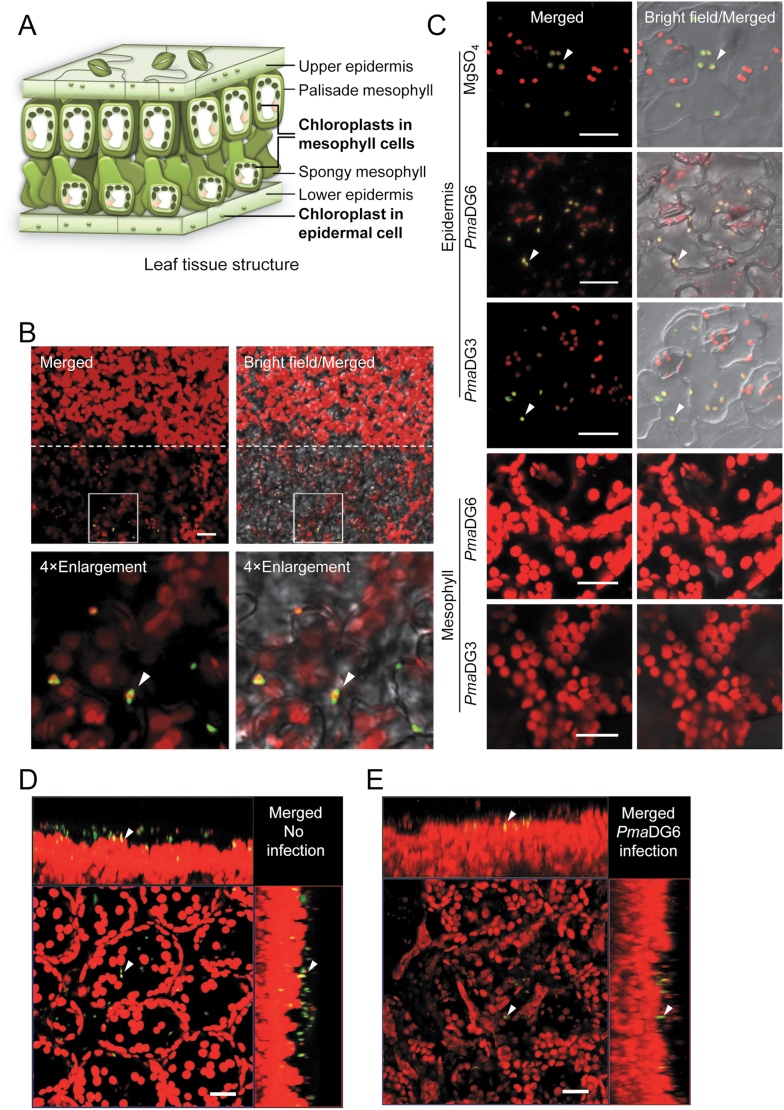
Epidermal cell-specific accumulation of ALD1:GFP fusion proteins after DEX treatments of leaves with or without infection. (A) Diagram of leaf structure and chloroplasts in different cell types. The size of chloroplasts in upper and lower epidermal cells is much smaller than that of the chloroplasts in mesophyll cells. (B–E) Laser scanning confocal micrographs of DEX-induced ALD1:GFP fusion protein in leaves of Arabidopsis transgenic lines *pDEX::ALD1* #6 or #10. GFP fluorescence is shown in green and chlorophyll autofluorescence is shown in red. Scale bar=20 μm. (B) Expression and localization of DEX-induced ALD1:GFP fusion protein in the abaxial side of a half peeled leaf in *pDEX::ALD1* #10. Above the dotted line are the mesophyll cells after removing the lower epidermis, while the part below is the original leaf with epidermis. Leaves were soaked in 30 μM DEX for 2 d before peeling. Four times (4×) enlarged images of selected insets are shown in the lower panels. Single layer scanning images were used. Similar results were observed in three independent experiments (*n*≥6 biological replicates for each experiment). (C) ALD1:GFP fusion protein in the epidermis or mesophyll of line *pDEX::ALD1* #6 after pathogen *Pma* infection. Leaves were first sprayed with 30 μM DEX for 2 d, then inoculated by *Pma*DG6, *Pma*DG3 (OD_600_=0.01), or 10 mM MgSO_4_ for 18 h. Epidermal strips were peeled from the abaxial surface of the leaf, and the mesophyll layer was from the corresponding peeled region. Maximum intensity projections of *Z*-series images are used for epidermis data. Similar results were observed in two independent experiments (*n*≥6 biological replicates for each experiment). (D and E) Maximum intensity projections of the leaf with orthogonal projections to the *XY*, *XZ*, and *YZ* planes. Arrows indicate the same plastid. Leaves of line *pDEX::ALD1* #6 were pre-treated with perfluorodecalin. Similar results were observed in two independent experiments (*n*≥6 biological replicates for each experiment). (D) Leaves of a 28-day-old plant were infiltrated with 30 μM DEX for 2 d. Images were taken from the adaxial surface of the leaf before infection. (E) Leaves of a 24-day-old plant were sprayed with 60 μM DEX for 1.5 d, and then infiltrated with *Pma*DG6 (OD_600_=0.01) for 18 h. Images were taken from the abaxial surface of the leaf after infection.

To more precisely distinguish between mesophyll and epidermal cells, we employed a leaf peeling approach to separate the epidermal and mesophyll layers. A schematic of the leaf cell layers is shown in Fig. 2A. ALD1:GFP signals co-localized with chloroplast autofluorescence signals in epidermal cells, whereas no GFP signal was detected in mesophyll cells. This epidermal cell-specific accumulation of ALD1 was observed in leaves soaked in (Fig. 2B) or sprayed with DEX (Fig. 2C). Both pavement cells and guard cells were found to accumulate ALD1:GFP signals.

To further study the cell type-specific accumulation of ALD1:GFP, we analyzed *Z*-stack images of plant leaves by maximum intensity projections with orthogonal projections to the *XY*, *XZ*, *YZ* planes and 3D reconstructions to better illustrate the location. ALD1:GFP signals overlapped with the chloroplast autofluorescence mainly in the epidermal cells layer, as shown in Fig. 2D, Supplementary Fig. S2, and Supplementary Video S1. In contrast, the transgenic marker line pt-gk (CS16266, ABRC) containing GFP localized to plastids (Nelson *et al.*, 2007) showed GFP signals that overlapped with chloroplast autofluorescence in both epidermal and mesophyll cells (Supplementary Fig. S2; Supplementary Video S2). As expected, WT plants showed no GFP signal (Supplementary Fig. S2; Supplementary Video S3).

**Fig. 3.**
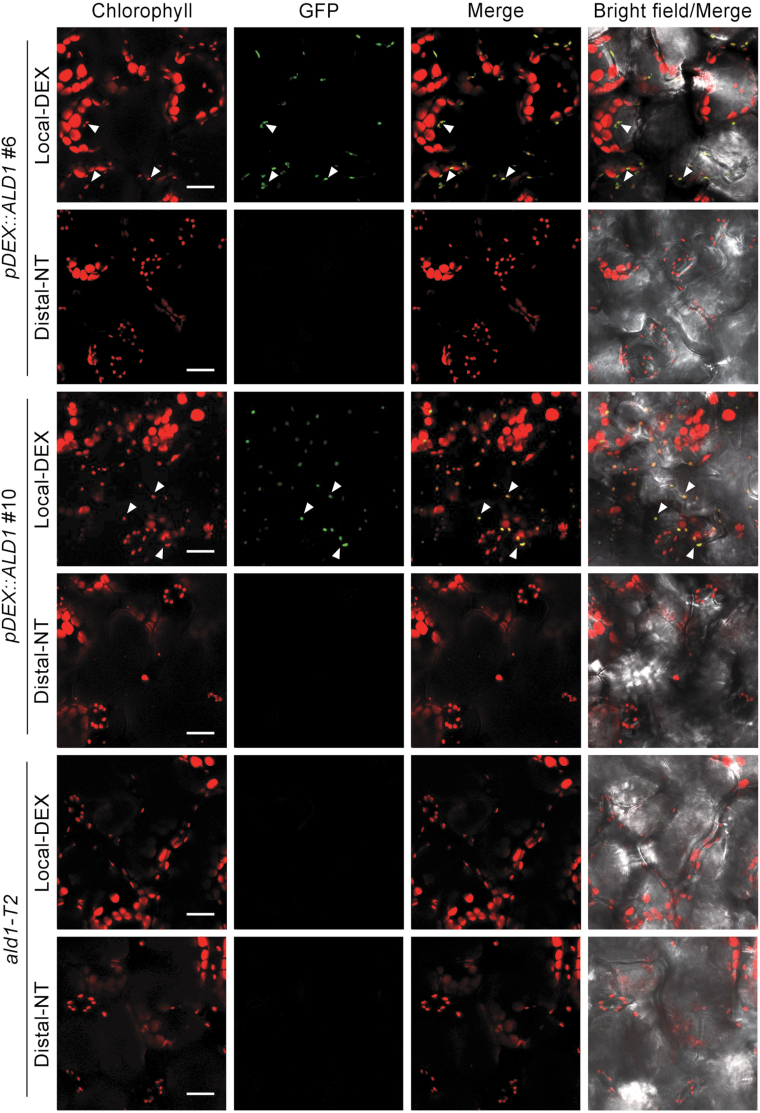
Accumulation of ALD1:GFP 4 d post-treatment only in leaves directly painted with DEX. Confocal *Z*-series maximum intensity projection showing images of DEX-inducible ALD1:GFP fusion protein in transgenic *pDEX::ALD1* lines #6 and #10. DEX-treated local leaves (Local-DEX) and no-treatment distal leaves (Distal-NT) were collected at 4 d after 30 μM DEX painting on local leaves. The *ald1-T2* mutant was used as a negative control. Chlorophyll autofluorescence is shown in red, and GFP fluorescence is shown in green. Scale bar=20 μm. Biological replicates: local leaves, *n*=6; distal leaves, *n*=3. White arrowheads indicate the representative chloroplasts and ALD1:GFP signals showing co-localization in the merged images. Similar results were observed in other independent experiments after 2 d DEX painting as shown in Supplementary Fig. S3.

To analyze if infection affected the cell type-specific accumulation of ALD1, we studied the distribution of ALD1:GFP in peeled epidermal and mesophyll cells after DEX treatment and infection with the virulent bacterial strain *Pma*DG3, which is derived from *P. cannabina* pv. *alisalensis* (Bull *et al.*, 2010) formerly called *P. syringae* pv. *maculicola* ES4326, and the isogenic avirulent strain carrying *avrRpt2* (*Pma*DG6) that we typically use to activate SAR. As shown in Fig. 2C and E and in Supplementary Fig. S2, ALD1:GFP signals only co-localized with the small epidermal chloroplasts during both infection conditions. Both *pDEX::ALD1* lines showed GFP signal only in epidermal cell chloroplasts of the local leaves at 4 d (Fig. 3) or 2 d (Supplementary Fig. S3) after DEX painting. ALD1:GFP signals were not detectable in any distal leaves after DEX treatment and infection of lower leaves.

These results indicate that the expression system was not leaky, as DEX treatment resulted in *ALD1:GFP* expression only at the sites of application. Additionally, ALD1:GFP was only detected in the chloroplasts of epidermal cells directly treated with DEX, and different DEX treatment approaches including painting, spraying, soaking, and pressure infiltration showed the same outcome. Furthermore, pathogen infections did not alter the cell type-specific pattern of ALD1:GFP accumulation.

### Epidermal-enriched ALD1:GFP accumulation fully restores local responses

**Fig. 4.**
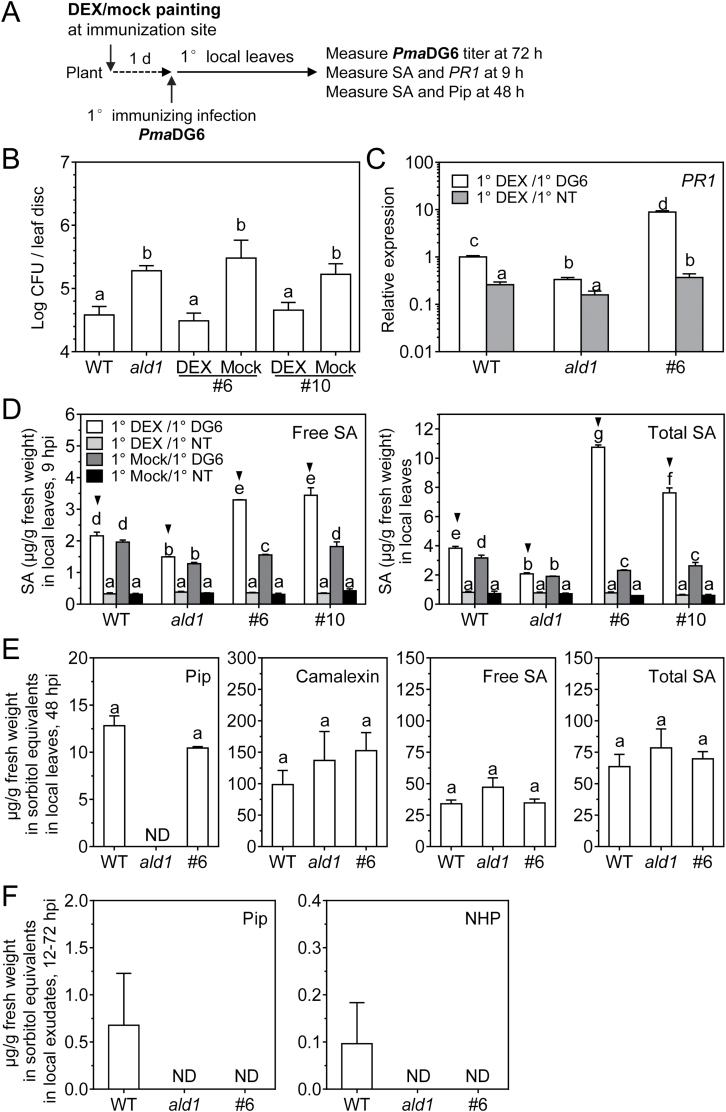
ALD1 accumulation at the site of infection fully restores defense responses in local leaves. (A) Treatment schemes in local leaves in (B–E). Local leaves (the third to fifth leaves) were painted with DEX (30 μM) or mock treated for 1 d, and then inoculated with *Pma*DG6. The primary (1°) local leaves were then collected at the indicated times for further analysis. (B) Titer of *Pma*DG6 in local leaves of the WT, *ald1-T2* (*ald1*), and DEX- or mock-treated transgenic *pDEX::ALD1* lines #6 and #10. Colony-forming unit (CFU) number was measured in local leaves on day 3 after infection with *Pma*DG6 (OD_600_=0.0001). Error bars indicate the SEM of eight biological replicates. The experiment was repeated three times with similar results. Another experiment that employed DEX spraying also showed similar results. (C) *PR1* gene expression level in DEX- (30 μM) painted local leaves at 0 h (no treatment, NT) and 9 h after *Pma*DG6 (DG6, OD_600_=0.01) infection in the indicated genotypes: wild type (WT), *ald1-T2* (*ald1*), and *pDEX::ALD1*#6 (#6). Error bars indicate the SEM from at least two biological replicates and three technical replicates. Each biological replicate consists of 6–9 leaves from at least three plants. The experiment was repeated twice with similar results. (D) Endogenous salicylic acid (SA) levels in local leaves were measured by HPLC in the indicated genotypes. DEX- (30 μM) or mock-painted local leaves were collected at 0 h (no treatment, NT) or 9 h after *Pma*DG6 (DG6, OD_600_=0.01) infection. Free SA is shown in the left panel, and total SA is shown in the right panel. Error bars indicate the SEM of at least three biological replicates. Each biological replicate consists of 6–9 leaves from at least three plants. (E) Defense-related metabolite levels measured by GC-MS in local leaves of the indicated genotypes after 48 h infection. Error bars indicate the SEM from four biological replicates. (F) Pip and NHP levels in petiole exudates are not rescued in *pDEX::ALD1* plants. Plants at ~4 weeks old of the WT, *ald1*, and *pDEX::ALD1*#6 (#6) were sprayed with 30 μM DEX for 1 d before infection. Petiole exudates were collected during 12–72 h post-local inoculation of the SAR-inducing *Pma*DG6 strain (OD_600_=0.01). Metabolite levels measured by GC-MS. Results are the average with the SE from six biological replicates. Each biological replicate contains 12 leaves in 1.4 ml of 1 mM Na_2_-EDTA (pH 8.0) solution. Different letters indicate statistically significant differences (*P*<0.05, ANOVA, Fisher’s LSD test). ND, not detected; hpi, hours post-infection.

To test if epidermal-enriched ALD1:GFP could rescue local responses in the *ald1* mutant background, we measured the pathogen growth and defense signaling in local leaves directly treated with DEX, as indicated in the scheme of Fig. 4A. We infiltrated leaves with the avirulent strain *Pma*DG6, as this strain was used in subsequent experiments to trigger SAR (see sections below). Growth of *Pma*DG6 was inhibited in the DEX-treated leaves of *pDEX::ALD1* lines #6 and #10 (Fig. 4B). In contrast, without DEX treatment, *pDEX::ALD1* lines were as susceptible as the *ald1* mutant.

Next, we measured defense signaling outputs at the indicated times post-infection, including *PR1* gene expression, SA, Pip, and levels of other defense-related metabolites using the set-up shown in Fig. 4A. The time point for analyzing induced *PR1* gene expression (Fig. 4C) and SA levels (Fig. 4D) was 9 h (early response after infection), as values measured at this time point are known to show significant differences in signaling outputs between WT and *ald1* plants (Song *et al.*, 2004*b*; Cecchini *et al.*, 2015*a*). As shown in Fig. 4C, D, *PR1* transcript and SA levels in DEX-treated and infected *pDEX::ALD1* plants were significantly higher at 9 h when compared with the WT and *ald1*. By 48 h post-infection of DEX-treated plants, *pDEX::ALD1* #6 showed no differences in SA levels compared with other genotypes (Fig. 4E). The SA levels in uninfected leaves were similar in all genotypes with or without DEX treatment. The antimicrobial compound camalexin was only detectable in plant extracts prepared 48 h after infection, and showed no difference among all genotypes. Pip was not detectable at 9 h or 18 h post-*Pma*DG6 infection in all genotypes. At 48 h after infection, the Pip level was restored in *pDEX::ALD1* #6 when compared with the WT, and it was still not detectable in *ald1*, as expected (Fig. 4E). NHP was not detectable in the leaf extracts analyzed.

Because Pip/NHP are proposed SAR-priming systemic signals produced by ALD1 (Návarová *et al.*, 2012; Hartmann *et al.*, 2018), we analyzed if ALD1 activity in epidermal cells of local leaves generated mobile Pip or NHP. To do this, we collected petiole exudates of DEX-treated *pDEX::ALD1* line #6 for 3 d after *Pma*DG6 infection. Pip and NHP were detected in the WT exudates, but were not detectable in exudates from DEX-treated line #6 or *ald1* mutant plants (Fig. 4F).

Together our data indicate that epidermal production of ALD1 can fully restore disease resistance, SA signaling, and defense responses during a local infection. However, epidermal-enriched ALD1 did not result in the mobilization of the ALD1-produced metabolites Pip and NHP into the vascular fluid.

### Epidermal-enriched ALD1:GFP produced only at the immunization site rescues the response gain of SAR in *ald1*

**Fig. 5.**
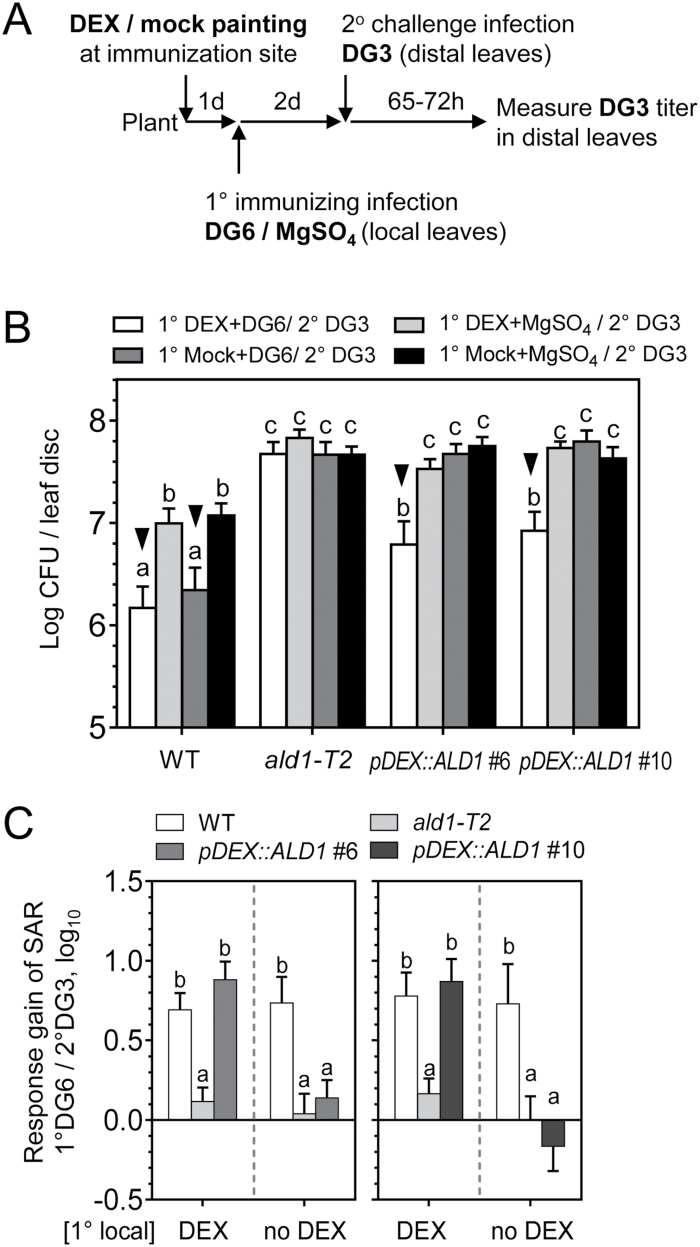
Specific expression of ALD1 at the immunization site restores SAR in distal leaves. (A) Treatment schemes for specific expression of ALD1 at the immunization site during SAR establishment. Typically, local leaves (1°, the third to fifth leaves) were painted with 15–30 μM DEX or mock solution prior to SAR-triggering primary infection of an avirulent strain *Pma*DG6 (DG6, OD_600_=0.01) or 10 mM MgSO_4_. Then distal leaves (2°, the sixth to eighth leaves) without DEX treatment were inoculated with a virulent *Pma*DG3 (DG3, OD_600_=0.0002) for the secondary infection. The quantification of DG3 growth in distal leaves was determined ~65–72 h later. (B) Titer of DG3 in distal leaves of the indicated genotypes. The number of colony-forming units (CFUs) of DG3 was measured in distal leaves. Error bars indicate the SEM of eight biological replicates (from eight plants). The result is representative of five independent experiments with similar results. Black triangles indicate SAR establishment under the corresponding treatment conditions. (C) Response gain of SAR associated with immunizing infection by 1° DG6 in local leaves with or without DEX treatment. Data for the line *pDEX::ALD1* #6 (left panel) are the average of 2–3 experiments (DEX, three times; no DEX, twice), while data for the line *pDEX::ALD1* line #10 (right panel) are the average of 1–2 experiments (DEX, twice; no DEX, once). Error bars indicate average uncertainties from the indicated experiments. Different letters indicate statistically significant differences (*P*<0.05, ANOVA, Fisher’s LSD test).

The DEX painting approach enables the production of functional ALD1 only where DEX is applied to *ald1* plants carrying *pDEX::ALD1*. Therefore, we tested whether accumulation of ALD1:GFP at the immunization site could restore SAR. Figure 5A shows the timing of the steps in the SAR experiment. Briefly, ALD1 expression was induced by DEX painting of lower (local) leaves. After 1 d, the same lower leaves were inoculated with strain *Pma*DG6. Two days after this primary infection, a secondary infection of distal leaves was made with virulent *Pma*DG3 to assess SAR. As shown in Fig. 5B, immunization with *Pma*DG6 together with epidermal accumulation of ALD1:GFP in the *pDEX::ALD1* lines #6 and #10 restored SAR in the distal leaves, observed as decreased growth of *Pma*DG3. DEX treatment alone did not alter the distal leaf pathogen growth in WT and *ald1* plants. As expected, the WT established SAR, whereas the *ald1* mutant was hypersusceptible to pathogen infection and lacked SAR using any treatment condition. Symptoms of representative infected distal leaves are shown in Supplementary Fig. S4. The use of another avirulent strain of *P. cannabina* pv. *alisalensis* carrying *avrRpm1* (*Pma*DG34) to induce SAR yielded similar results (Supplementary Fig. S5A, B).

Interestingly, the absolute level of bacteria in distal leaves of *pDEX::ALD1* lines during SAR was still higher than that of the WT in four of five independent experiments. To assess the activation level of SAR in different genotypes, we calculated the response gain of SAR due to primary infection (see the Materials and methods for the detailed response gain calculation and propagation of uncertainties). In the *Pma*DG6-triggered SAR, the response gain of local DEX-treated *pDEX::ALD1* lines #6 and #10 showed no significant difference when compared with that of the WT (Fig. 5C). As expected, the response gain of SAR in the SAR-deficient *ald1* mutant was markedly lower and the value was close to 10^0^ (no gain). Importantly, without local DEX treatment, the response gains of *pDEX::ALD1* lines #6 and #10 plants were close to that of *ald1* mutant. Similar results were observed when SAR was triggered by *Pma*DG34 (Supplementary Fig. S5C).

Together, these results indicate that the epidermal-enriched accumulation of ALD1 at local immunization sites can restore SAR. Although the absolute levels of the pathogen in *pDEX::ALD1* lines was still higher when compared with the WT induced for SAR, the response gain of SAR was similar to that of the WT.

### Local epidermal-enriched ALD1:GFP accumulation can induce SAR-associated defense priming

**Fig. 6.**
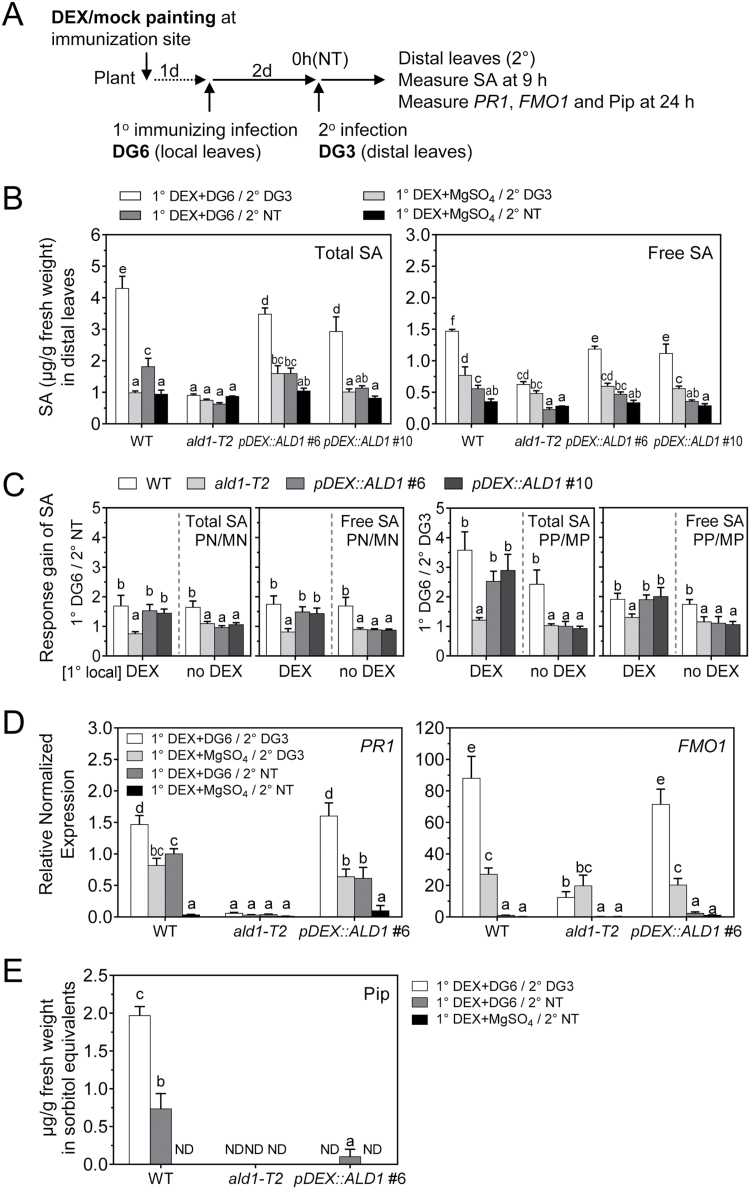
ALD1 accumulation at the site of an immunizing infection restores many distal leaf defenses but not Pip accumulation. (A) Treatment schemes for SA measurement in distal leaves in (B–D). After 1° DEX (30 μM) painting for 1 d, local leaves were infection by *Pma*DG6 (OD_600_=0.01) or 10 mM MgSO_4_. Then after 1° immunization infection for 2 d, distal leaves (without DEX treatment) were collected at 0 h (NT) or the indicated times after 2° challenge infection with *Pma*DG3 (OD_600_=0.01). (B) SA levels in distal leaves of the indicated genotypes induced by immunizing infection before 2° infection (2° NT) and after 2° infection (2° DG3). SA levels were measured by HPLC in different genotypes after treatments. After 1° immunization infection for 2 d, distal leaves (without DEX treatment) were collected at 0 h (NT), or 9 h after 2° challenge infection with *Pma*DG3. Error bars indicate the SEM from four biological replicates. Each biological replicate consists of 6–9 leaves from three plants. (C) Response gain of SA in distal leaves of the indicated genotypes due to 1° *Pma*DG6 and 2° NT (1° DG6/2° NT, PN/MN), or due to 1° *Pma*DG6 and 2° *Pma*DG3 (1° DG6/ 2° DG3, PP/MP), corresponding to the SA data in (B). Meaning of symbols: PN, 1° *Pma*DG6 and 2° no treatment; MN, 1° MgSO_4_ and 2° no treatment; PP, 1° *Pma*DG6 and 2° *Pma*DG3; MP, 1° MgSO_4_ and 2° *Pma*DG3. (D) Expression levels of defense-related genes *PR1* and *FMO1* in distal leaves of the indicated genotypes induced by 1° immunizing infection and 2° challenge infection. The distal leaves were collected with no treatment (2° NT) or 2° *Pma*DG3 for 24 h. *ACTIN* was used as an internal reference. Error bars indicate the SEM from three biological replicates and two to three technical replicates. (E) Pip levels in distal leaves of the indicated genotypes measured by GC-MS. After 2 d immunization infection by *Pma*DG6, distal leaves (without DEX treatment) were collected at 0 h (NT), or 24 h after 2° infection with *Pma*DG3 (DG3). Error bars indicate the SEM from three biological replicates. Each biological replicate consists of 6–9 leaves from three plants. ND, not detected. Different letters indicate statistically significant differences (*P*<0.05, ANOVA, Fisher’s LSD test). For (C), the comparisons are within the DEX group or no DEX group, respectively.

To assess SAR-associated defense priming triggered by local infection in plants that accumulate ALD1:GFP only at the immunization site, we tested signaling outputs during SAR in the distal leaves, including the accumulation of SA, Pip, and defense-related genes (Fig. 6A). DEX-induced local ALD1:GFP accumulation combined with the primary immunization boosted the accumulation of SA triggered by secondary infection with *Pma*DG3 in distal leaves of both *pDEX::ALD1* #6 and #10 lines, similar to what was observed in the WT (Fig. 6B). In contrast, the *ald1* mutant showed no induction of SA level in either immunized or non-immunized plants. As expected, this SAR-associated priming effect on SA biosynthesis was not observed when DEX was not applied to *pDEX::ALD1* lines #6 and #10 plants (Supplementary Fig. S6). Without primary (local) ALD1 expression or primary immunizing infection, *pDEX::ALD1* lines showed no induction of SA, similar to the *ald1* mutant plant phenotype.

 To assess the quantitative difference of SA induction levels in distal leaves, we calculated the response gain of SA accumulation due to primary immunization infection. As shown in Fig. 6C, in *pDEX::ALD1* lines #6 and #10 plants with DEX treatment and primary local immunization by *Pma*DG6, the response gain of SA in DEX-non-treated distal leaves showed no significant difference from the WT before or after secondary infection. Without DEX treatment at the primary immunization site, the SA response gain of *pDEX::ALD1* plants was as low as in the *ald1* mutant.

We also investigated whether ALD1:GFP accumulation at primary immunization sites treated with *Pma*DG6 altered *PR1* and/or *FMO1* transcript accumulation in distal leaves. As assessed by qPCR in Fig. 6D, accumulation of ALD1:GFP in *pDEX::ALD1* line #6 at primary immunization sites infected with *Pma*DG6 restored the systemic induction and SAR-associated priming of *PR1* and *FMO1* transcripts, which is similar to what was observed in WT plants. In the SAR-deficient *ald1* mutant, the *PR1* expression levels were not altered by any infection, while the *FMO1* level was not induced by immunization infection and showed no priming effect. However, the *PR1* expression levels in the WT and *pDEX::ALD1* line #6 were induced in distal tissue after primary infection alone and also after secondary infection, while *FMO1* expression levels were induced only after secondary infections.

We next analyzed the accumulation of Pip in distal leaves after primary infection of local DEX-treated leaves. As shown in Fig. 6E, Pip accumulation in distal leaves of the WT was induced after primary infection, and then significantly up-regulated after secondary infection (24 h). However, much less Pip accumulated in *pDEX::ALD1* #6 than that observed in the WT before and after secondary infection, and was not detected in most samples. NHP was not detected in distal leaves of different genotypes under different treatment conditions, which is similar to our analysis of local leaves. This agrees with our finding that Pip and NHP did not accumulate in petiole exudates of *pDEX:ALD1* #6 plants (Fig. 4F). Nevertheless, in *pDEX::ALD1* #6 with primary infection of local DEX-treated leaves, the SA accumulation in distal leaves after secondary infection (24 h) was restored to a level similar to that in the WT (Supplementary Fig. S7).

Taken together, our results suggest that epidermal-enriched ALD1 accumulation at primary immunization sites restores SAR-associated priming of SA and defense-related gene expression in distal leaves. Interestingly, SAR restoration occurs without appreciable Pip or NHP accumulation in secondary distal leaves, implicating additional signal(s) as important in SAR activation.

### ALD1:GFP accumulation exclusively at the secondary (distal) infection site cannot restore SAR

**Fig. 7.**
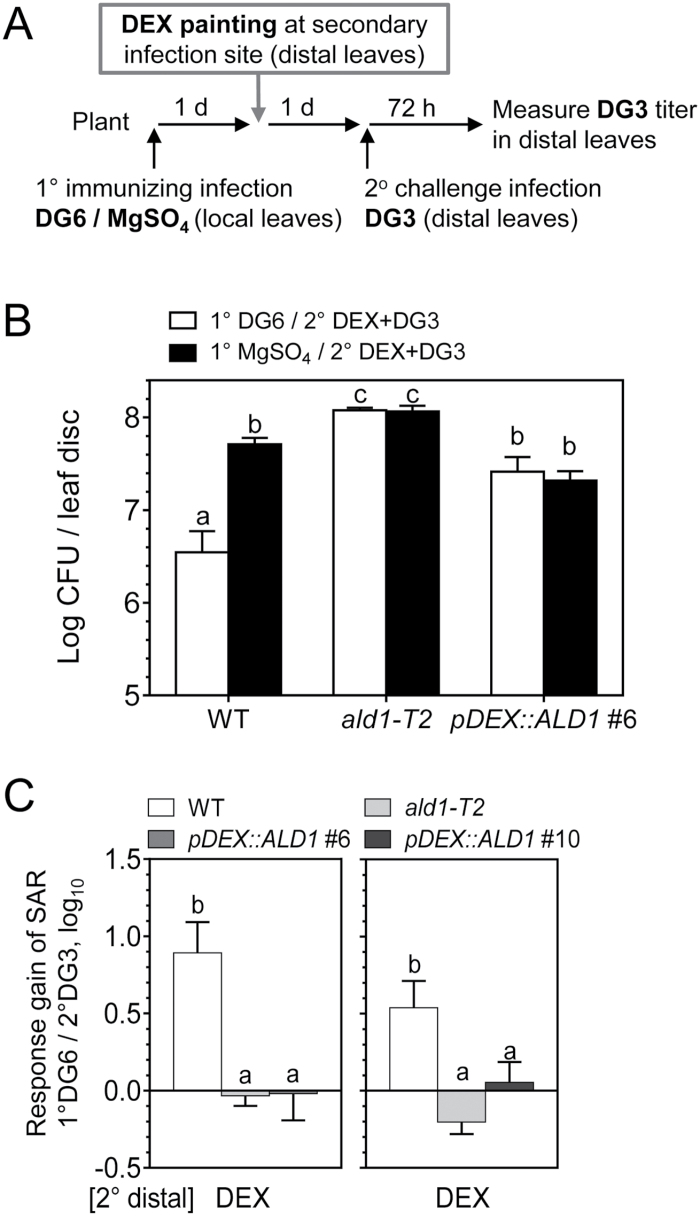
ALD1 accumulation at the 2° distal challenge site does not restore SAR. (A) Treatment scheme for specific expression of ALD1 at the 2° challenge infection site in SAR. Local leaves (the third to fifth leaves) were infiltrated with DG6 (OD_600_=0.01) or 10 mM MgSO_4_ during immunizing infection. Then distal leaves (the sixth to eighth leaves) were painted with DEX at 1 d prior to 2° challenge infection by DG3 (OD_600_=0.0001). The quantification of DG3 growth in distal leaves was measured 72 h later. (B) SAR response in distal leaves of the WT, *ald1-T2*, and *pDEX::ALD1* #6 painted with 3 μM DEX. Growth of DG3 was measured in distal leaves. Error bars indicate the SEM from eight biological replicates. Another independent experiment with 15 μM DEX painting on distal leaves showed similar results. (C) Response gain of SAR in DEX-painted distal leaves due to 1° immunizing infection by DG6 (1° DG6/2° DG3). Results for *pDEX::ALD1* #6 (left panel) and #10 (right panel) are each the average of two independent experiments. Different letters indicate statistically significant differences (*P*<0.05, ANOVA, Fisher’s LSD test).

Considering that ALD1 at the secondary infection sites (distal leaves) may also contribute to defense responses, we analyzed whether ALD1:GFP accumulation only at the secondary infection site could rescue SAR. To do this, upon the primary immunizing infection by *Pma*DG6, only the distal leaves (secondary infection site) were treated with 3 μM or 15 μM DEX and then inoculated with *Pma*DG3 (Fig. 7A). The timing and level of DEX treatment relative to pathogen treatment were used to ensure timely ALD1 induction, similar to when systemic ALD1 is detected in secondary tissue during infection of the WT. Furthermore, this set-up minimized possible side effects of long-term DEX exposure to leaves (Kang *et al.*, 1999).

As shown in Fig. 7B, when ALD1 was only present in distal leaves of *pDEX::ALD1* plants, no significant difference in pathogen growth was detected between plants with or without the primary immunizing infection with *Pma*DG6. The *pDEX::ALD1* plants showed lower pathogen growth when compared with the growth in *ald1.* Nevertheless, when ALD1:GFP accumulated in distal (secondary site) leaves, they still could not establish SAR when compared with the WT. Most importantly, the response gain of SAR in such secondary DEX-treated *pDEX::ALD1* lines was similar to that of *ald1*, and significantly lower than that of the WT (Fig. 7C).

Therefore, ALD1:GFP that accumulates only at the secondary infection site cannot restore SAR, at least under the experimental set-up that we used. When expressed >24 h after the onset of the local infection, ALD1:GFP at the secondary infection site appears to perform its role in basal defense, but not to contribute to the response gain of SAR. Furthermore, this experiment also shows that the predominantly epidermal production of ALD1 complements the susceptibility to the virulent strain *Pma*DG3, supporting the view that epidermal-enriched ALD1 confers basal resistance to *P. syringae*.

## Discussion

**Fig. 8.**
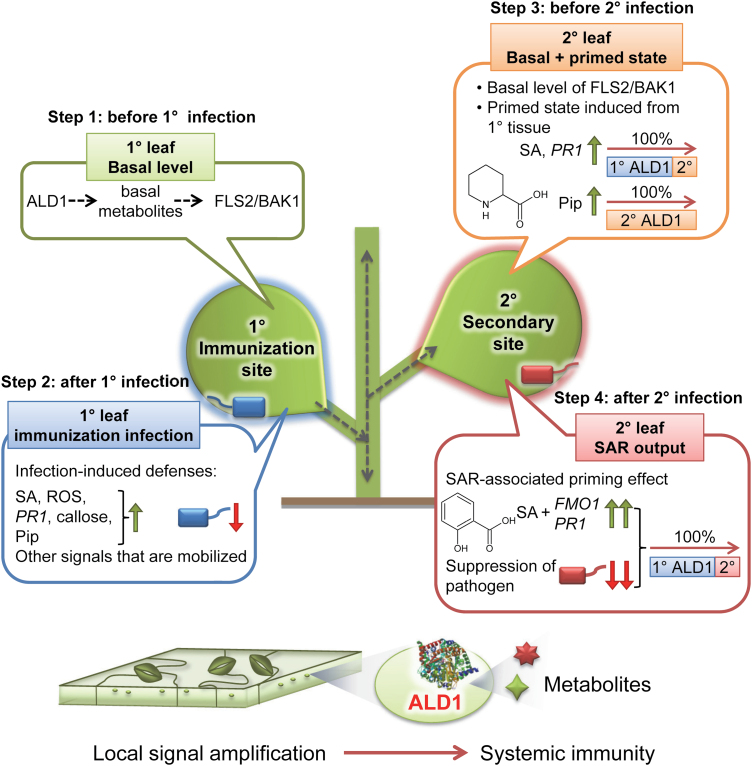
Proposed ALD1 site of action model during local defenses and different stages of SAR. Step 1: basal defense status before 1° infection. ALD1 mainly regulates the basal level of pattern recognition receptor complex FLS2/BAK1 possibly mediated by non-Pip basal metabolites (Cecchini *et al.*, 2015*a*). This study also shows that ALD1 predominantly in epidermal cells is sufficient to control infections with virulent and avirulent bacteria. Step 2: ALD1 at the 1° immunization site in epidermal cells is sufficient for the local defense responses (pathogen suppression, SA and ROS accumulation, callose deposition, defense gene expression, Pip biosynthesis, and other mobile immune signals for SAR establishment). Step 3: primed state at the distal leaf before 2° infection. ALD1 at the distal leaf is indispensable for most of the biosynthesis of Pip. 1° ALD1 in the epidermal cells at the immunization site contributes to controlling the majority of the accumulation of SA and defense genes (such as *PR1*) at the distal leaf. Step 4: 1° and 2° ALD1 expression regulate SAR output after 2° infection at the distal leaf. The local ALD1 predominantly in the chloroplasts of epidermal cells is sufficient to restore the systemic immunity. Blue, SAR-triggering bacteria at the 1° immunization site; red, virulent pathogenic bacteria infection at the 2° infection site. The ALD1 protein structure model (Sobolev *et al.*, 2013) was downloaded from https://www.rcsb.org/structure/4FL0.

ALD1 is an aminotransferase that is crucial for achieving full disease resistance to *P. syringae*. It catalyzes a transamination reaction for the biosynthesis of several defense-related signals and is needed for both inducible local and systemic defenses (Song *et al.*, 2004*a*, *b*; Návarová *et al.*, 2012; Cecchini *et al.*, 2015*a*; Chen *et al.*, 2018; Hartmann *et al.*, 2018; Hartmann and Zeier, 2019). This work establishes that ALD1 has a non-autonomous effect on pathogen growth and defense activation. By using plants in which ALD1 production was restricted to specific leaves and was detected in the epidermal cells of those leaves, we can make several new inferences. ALD1 in the epidermis can restore resistance to both virulent and avirulent strains of *P. syringae* (Fig. 4, 7). We used infiltration to infect the plants, which results in a large amount of bacteria that grow in association with mesophyll cells. Thus, our first inference is that either the epidermal cells secrete something ALD1 controlled that can restrict the bacteria associated with mesophyll neighbors, or the epidermal products move to mesophyll cells and/or cause signaling in the mesophyll to restrict bacterial growth. To confer SAR, ALD1 is only needed in the epidermis of the immunizing leaves (Figs 5, 6). Thus, our second inference is that either a direct product of the ALD1 pathway or a defense signal(s) produced by a component(s) in a regulatory loop with ALD1 can mobilize from the epidermis of the immunized leaves to the distal leaves. The bacterial growth patterns that we found highlight the requirement for ALD1 in immunized and distal leaves in order to achieve full disease resistance in all leaves. Thus, our third inference is that epidermal-enriched ALD1 has separable functions (possibly by producing different products) to affect basal disease resistance and also contribute to a full response gain seen during SAR. In Fig. 8, we summarize our findings about how epidermal-enriched ALD1 contributes to defense. It is possible that a small amount of experimentally undetectable ALD1:GFP accumulates in mesophyll cells of our plants, but the strong enrichment of the fusion protein in the epidermal cells in our experiments (Fig. 2, 3) supports the inferences above.

Epidermal cells are the first barrier of defense and the first contact point for interaction with the environment and microbes. Because ALD1 activity regulates basal FLS2 and BAK1 PAMP receptor/co-receptor protein levels (Cecchini *et al.*, 2015*a*), one possibility is that ALD1 specifically maintains the required levels of these PRRs in these first defensive cell layers. In agreement with this idea, it is known that PTI in the stomata or other epidermal cells helps prevent pathogen entrance (Melotto *et al.*, 2006; Zeng and He, 2010; Henty-Ridilla *et al.*, 2013; Kang *et al.*, 2014). Moreover, insect eggs are also recognized on the leaf surface, leading to induction of PTI and SAR (Hilfiker *et al.*, 2014). It is possible that the previously described defense amplification loop ALD1–PAD4–ICS1/SID2 (Song *et al.*, 2004*a*; Cecchini *et al.*, 2015*a*; Bernsdorff *et al.*, 2016) enhances ETI responses occurring in plant epidermal pavement and guard cells. Supporting this idea, it is known that epidermal cells are also a major target for pathogen effectors, some of which shape the epiphytic growth pattern of *P. syringae* (Lee *et al.*, 2012; Henry *et al.*, 2017). Therefore, it is possible that PTI and probably ETI pathways are boosted by ALD1 expression and play key roles in the plant epidermis. Importantly, *ALD1* transcripts have been detected in Arabidopsis epidermal cells, supporting its function in epidermal tissue (Yang *et al.*, 2008; Obulareddy *et al.*, 2013; Adrian *et al.*, 2015).

Recent studies suggest that the understudied epidermal plastids (and stromules) may play a central role in defense against pathogens (Caplan *et al.*, 2015; Cecchini *et al.*, 2015*b*; Beltrán *et al.*, 2018; Seguel *et al.*, 2018; Ding *et al.*, 2019; Toufexi *et al.*, 2019, Preprint). What does ALD1 do in epidermal plastids? Considering the reported enzymatic activity, it is probable that ALD1 plays a role in the synthesis of Pip/NHP and/or non-Pip basal defense metabolites that are required for both local and systemic defenses (Návarová *et al.*, 2012; Cecchini *et al.*, 2015*a*; Chen *et al.*, 2018; Hartmann *et al.*, 2018). Additionally, because ALD1 acts in a positive feedback loop together with the plastidic ICS1/SID2, it is possible that the epidermal plastids are a main source of SA for defense responses. In support of this idea, two other central regulators of SA production, EDS5 and PROHIBITIN3, preferentially accumulate in plastids of epidermal cells (as opposed to mesophyll cells) (Yamasaki *et al.*, 2013; Seguel *et al.*, 2018; Rekhter *et al.*, 2019; Torrens-Spence *et al.*, 2019). Moreover, SA biosynthesis genes are induced in epidermal stomata cells and may prevent pathogen entry (Zheng *et al.*, 2015).

We found that ALD1 that preferentially accumulates in epidermal cells at primary immunization sites restores SAR in the distal leaves (Fig. 5). As mentioned before, one idea is that the proposed SAR mobile signals Pip or NHP are mostly produced in the epidermis. However, ALD1 that locally accumulates mainly in the epidermis cannot restore Pip/NHP levels in distal leaves after primary infection (Fig. 6) and, moreover, Pip and NHP do not accumulate in the petiole exudates of such plants either (Fig. 4). Another possibility is that a Pip or NHP derivative that was not measured is a potential SAR signal generated by ALD1 in epidermal cells. In support of this idea, it was recently suggested that the derivative NHP-hexose is a probable mobile signal (Chen *et al.*, 2018; Hartmann *et al.*, 2018; Hartmann and Zeier, 2019). If this is true, NHP-hexose could be mostly produced in the epidermis. However, we were not able to detect NHP-hexose in any of the samples analyzed in this work. Alternatively, non-Pip-related metabolites may be epidermal cell-produced mobile signals (Cecchini *et al.*, 2015*a*) or there may be another mobile signal whose production relies indirectly on one or more ALD1-produced metabolites. Our findings also support the idea that the plant epidermis (and cuticle) may not only play a key role in the generation of the mobile SAR signal(s), but may also regulate or facilitate the movement/transport of signal(s) to the distal tissues (Xia *et al.*, 2012; Cecchini *et al.*, 2015*b*; Lim *et al.*, 2020).

ALD1 accumulation at the primary immunization site is able to rescue most of the SAR-associated responses. However, it cannot restore the absolute bacterial growth inhibition reached in WT plants nor the accumulation of Pip level in distal leaves (Figs 5, 6). Therefore, the pre-existing and/or infection-induced ALD1 at secondary infection sites contributes to the full level of disease resistance that can be achieved during SAR. One explanation is that the presence of ALD1 in systemic tissues allows for a full defense amplification loop where NHP, Pip, and/or non-Pip basal metabolites are necessary, as previously proposed (Cecchini *et al.*, 2015*a*; Hartmann *et al.*, 2018). This is consistent with our finding that ALD1 at primary immunization sites in epidermal cells cannot restore Pip/NHP increases at the secondary infection sites before infection.

Our experiments show that the SAR-specific function of ALD1 is in the primary leaves. In contrast, exudate experiments shown in Wang *et al.* (2018) might be interpreted as showing that ALD1 is dispensable in the primary leaves during SAR. In particular, *ald1* mutants could still generate active vascular exudates that conferred systemic immunity in WT plants. Petiole exudates are a powerful way to identify signal molecules. However, because petiole exudates are collected over time from many leaves, they can also concentrate signals that otherwise are insufficient during a natural infection to induce SAR. This is why it is possible that although *ald1* plants are SAR defective, they can still be used to identify biologically active compounds that may confer systemic immunity in the WT (either by directly moving or by inducing other compounds). As in Wang *et al.* (2018), our experiments support the conclusion that ALD1 contributes to disease resistance in systemic leaves. However, our experiments suggest that ALD1 contributes to basal resistance, not SAR, in these systemic leaves. Another explanation for the different conclusions about the role of ALD1 in primary leaves is that vascular exudates do not reflect the most important route of systemic movement of signal(s), which may be through an epidermal route.

In summary, we propose that ALD1 targeted to the epidermal plastids of the immunized leaves is sufficient to control local infections and activate SAR. Interestingly, other critical defense proteins for systemic resistance programs are also mainly detected in the widely underappreciated chloroplasts of epidermal cells in Arabidopsis (Yamasaki *et al.*, 2013; Cecchini *et al.*, 2015*b*), suggesting that epidermal cell plastids are closely related to systemic defenses. We speculate that the epidermis acts as a first conduit for cell to cell movement of signals, a skin-mediated systemic defense that may act via epidermal plasmodesmata (channels that can permit movement of molecules between cells). Future experiments tracking known and currently unknown signaling factors, and determining their mobilization in and between distinct cell layers/tissues during defense responses, are necessary to shed light on how important the epidermis is for plant immune signaling. In this sense, ALD1 could represent a paradigm for understanding the defensive functions of both epidermal cells and epidermal chloroplast during immunity.

## Supplementary data

The following supplementary data are available at *JXB* online.

Fig. S1. Accumulation of ALD1:GFP fusion proteins in whole leaves after DEX infiltration.

Fig. S2. Maximum intensity projections of GFP signals in leaves with orthogonal projections to the *XY*, *XZ*, and *YZ* planes.

Fig. S3. Accumulation 2 d post-treatment of ALD1:GFP only in leaves directly painted with DEX.

Fig. S4. Symptoms of representative distal leaves infected with *Pma*DG3 after SAR triggered by *Pma*DG6.

Fig. S5. Restoration of SAR in distal leaves when *Pma*DG34 is used as a primary immunizing infection of DEX-treated local leaves.

Fig. S6. Lack of restoration of SA accumulation in distal leaves during SAR without DEX treatments of *pDEX::ALD1* lines.

Fig. S7. GC-MS measurements confirm restoration of distal leaf accumulation of SA during SAR activation in *pDEX::ALD1* plants treated with DEX.

Table S1. Primer sequences.

Video S1. 3D view of *Z*-stack imaging of leaf of a *pDEX::ALD1* #6 plant expressing ALD1:GFP fusion protein after 30 μM DEX infiltration for 2 d (turn around the *x*-axis).

Video S2. 3D view of *Z*-stack imaging of leaf of the pt-gk (GFP) plastid marker line (turn around the *x*-axis).

Video S3. 3D view of *Z*-stack imaging of leaf of a WT plant (turn around the *x*-axis).

eraa609_suppl_Supplementary_Figures_S1-S7_Table_S1

eraa609_suppl_Supplementary_Video_1

eraa609_suppl_Supplementary_Video_2

eraa609_suppl_Supplementary_Video_3

## Data Availability

ABRC sequence data from this article can be found in the EMBL/GenBank data libraries under accession numbers At2g13810 (*ALD1*), At1g19250 (*FMO1*), and At2g14610 (*PR1*). The data supporting the findings of this study are available from the corresponding author, Jean T. Greenberg, upon request.
