# Supplementary material for: ALD1 accumulation in Arabidopsis epidermal plastids confers local and non-autonomous disease resistance
Source: J Exp Bot. 2021 Jan 19;72(7):2710–26. doi: 10.1093/jxb/eraa609 (PMC8006555; doi:10.1093/jxb/eraa609)
Supplement: eraa609_suppl_Supplementary_Figures_S1-S7_Table_S1 [file eraa609_suppl_supplementary_figures_s1-s7_table_s1.pdf]

Supplementary Figure S1

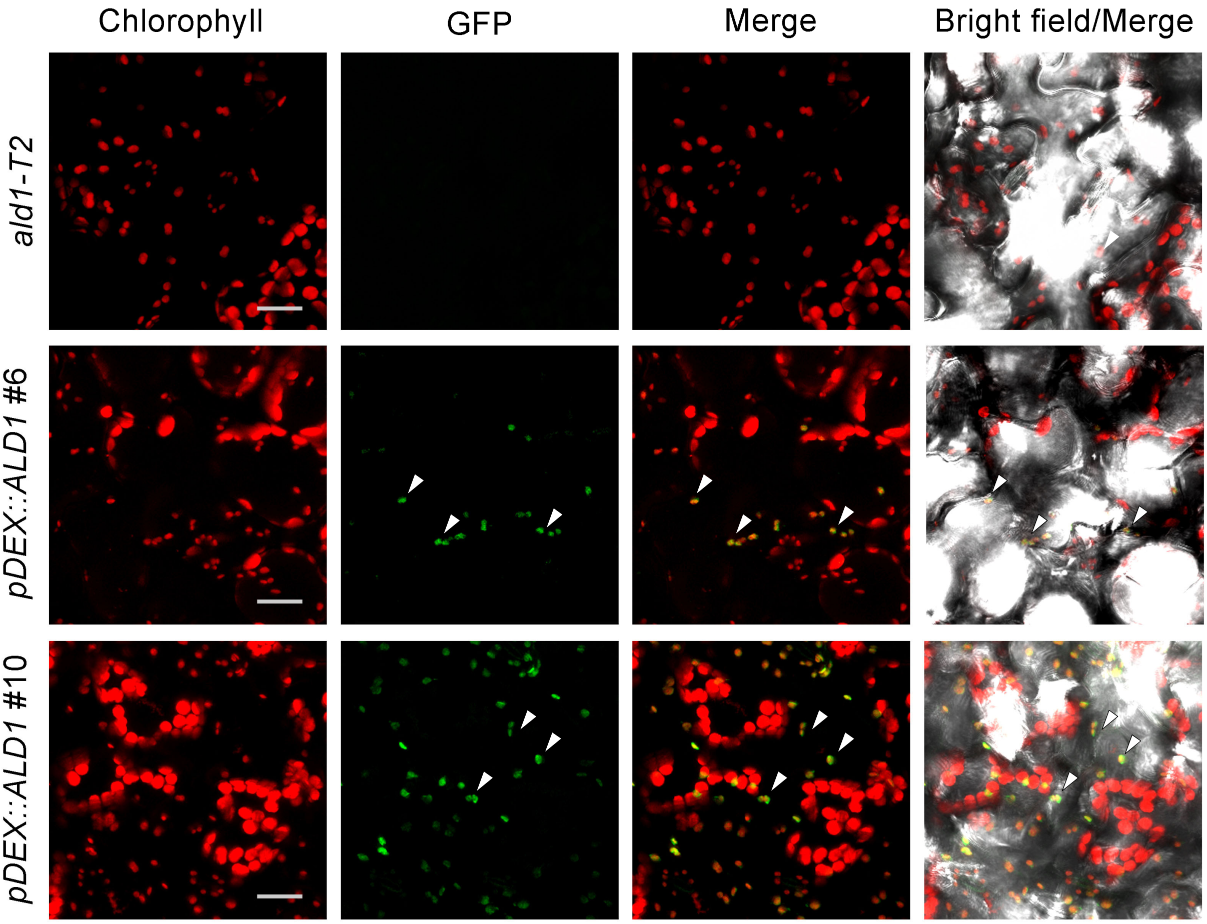

Fig. S1. Accumulation of ALD1:GFP fusion proteins in whole leaves after DEX infiltration.

Laser-scanning confocal images showed localization of DEX-inducible ALD1:GFP fusion protein in transgenic *Arabidopsis* lines *pDEX::ALD1* #6 and #10. Rosette leaves of about 25-28 days old plants were detected at 1 day after 60 μM DEX infiltration. *ald1-T2* mutant was used as negative control. Maximum intensity projections of Z series are shown. Chlorophyll auto-fluorescence is shown in red, and GFP fluorescence is shown in green. Scale bar, 20 μm. White arrowheads indicates the representative chloroplasts and ALD1:GFP signals showing co-localization in the merged images. Similar results were observed in four independent experiments. For each treatment there are more than 6 biological replicates.

Supplementary Figure S2

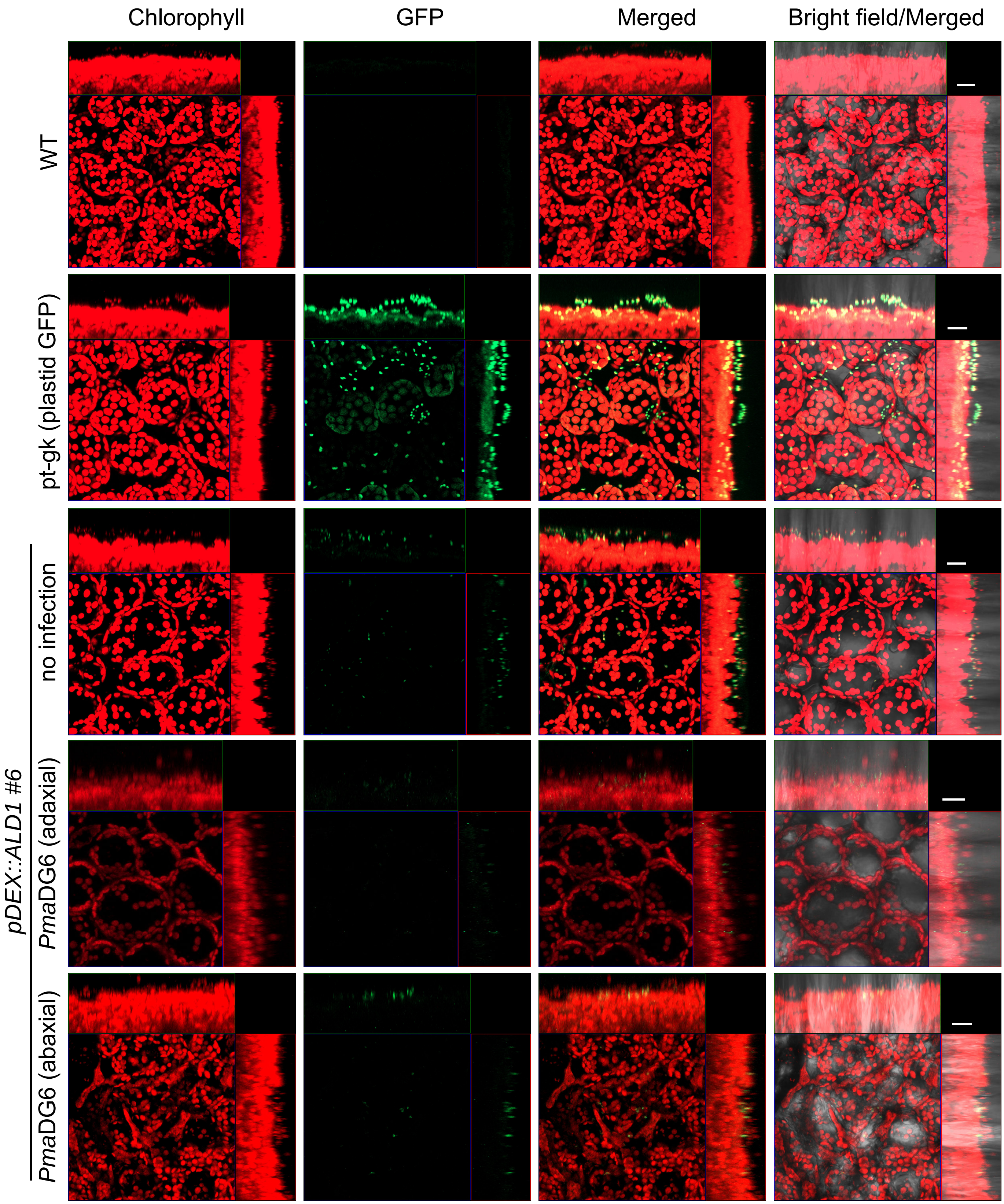

Fig. S2. Maximum intensity projections of GFP signals in leaves with orthogonal projections to the XY, XZ and YZ planes.

Projections of XY, XZ and YZ plane of the leaf were shown in the lower left, upper left and lower right of each image, respectively. All leaves were pretreated with perfluorodecalin. For WT and pt-gk plastid maker line (GFP), images were taken from adaxial surface of cotyledon of 7-d-old plants. For *pDEX::ALD1* #6 plants with no infection, leaves of 28-d-old plant were infiltrated with 30 μM DEX for 2 days, and images were taken from adaxial surface of leaf before infection (same treatment as Fig. 2D). For *pDEX::ALD1* #6 plants after *Pma*DG6 (OD_{600}=0.01) infection, leaves of 24-d-old plant were sprayed with 60 μM DEX for 1.5 days, and images were then taken at 18 h after infection from adaxial and abaxial surfaces (same treatment as Fig. 2E). Chlorophyll autofluorescence is shown in red, and GFP fluorescence is shown in green. Scale bar, 20 μm. Similar results were observed in two independent experiments. For each treatment there are more than 6 biological replicates.

Supplementary Figure S3

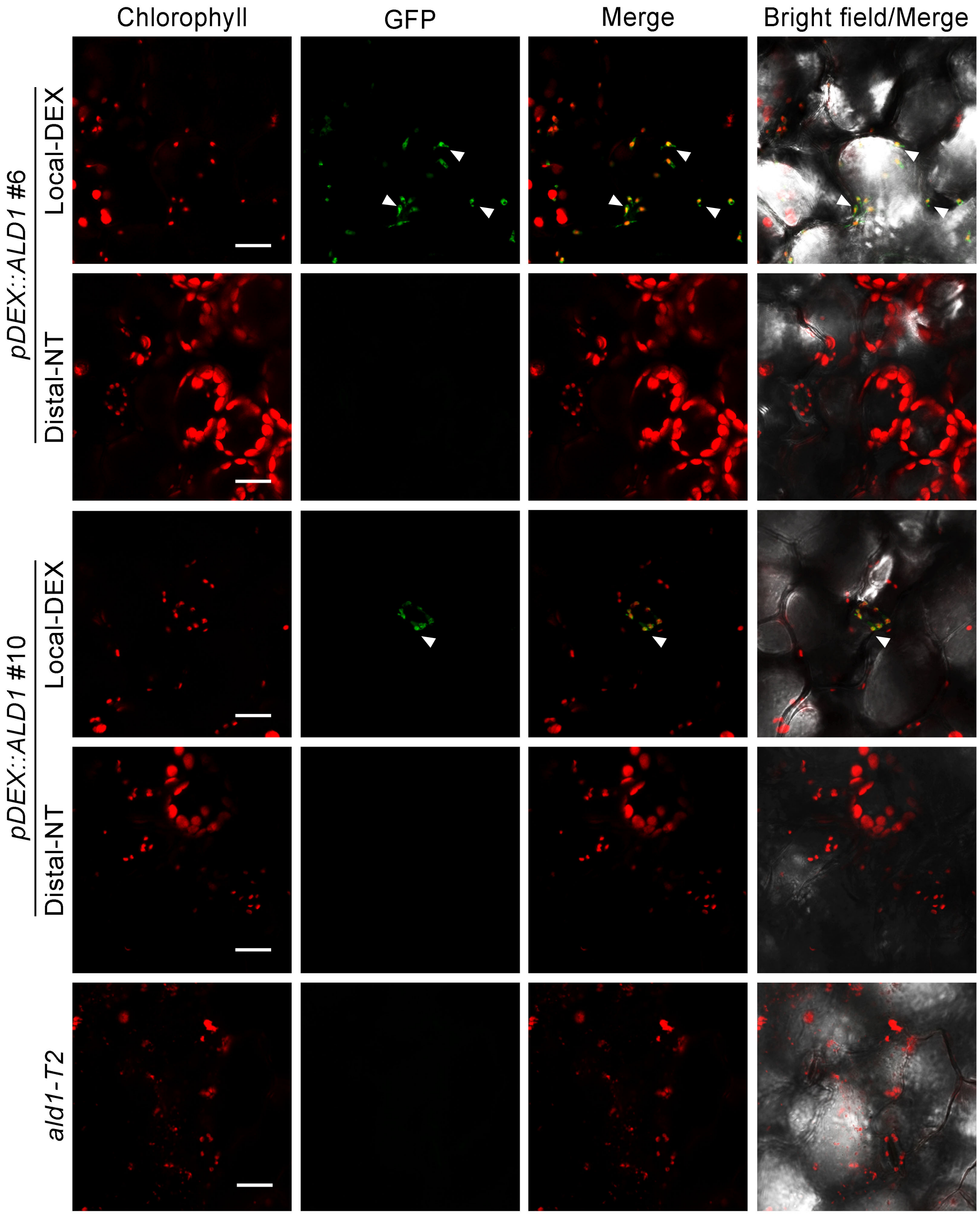

Fig. S3. Accumulation two days post-treatment of ALD1:GFP only in leaves directly painted with DEX.

Confocal Z-series maximum intensity projection showing images of DEX-inducible ALD1:GFP fusion protein in transgenic *pDEX::ALD1* lines #6 and #10. DEX-treated local leaves (Local-DEX) and no-treatment distal leaves (Distal-NT) were collected at 2 days after 30 μM DEX painting on local leaves. *ald1-T2* mutant was used as negative control. Chlorophyll autofluorescence is shown in red, and GFP fluorescence is shown in green. Scale bar, 20 μm. Biological replicates: local leaves, n=6; distal leaves, n=3. White arrowheads indicates the representative chloroplasts and ALD1:GFP signals showing colocalization in the merged images.

Supplementary Figure S4

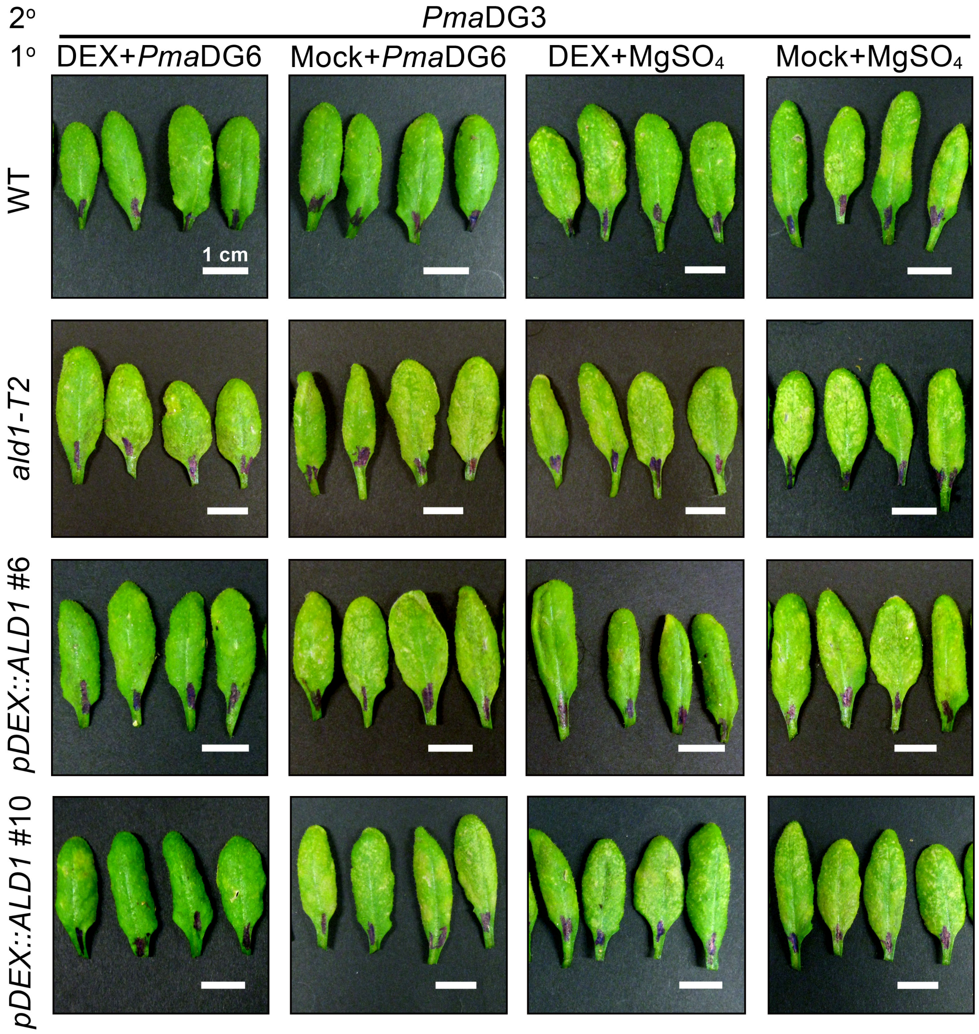

Fig. S4. Symptoms of representative distal leaves infected with *Pma*DG3 after SAR triggered by *Pma*DG6.

Local leaves (1°, the 3rd-5th leaves) were painted with 15-30μM DEX or mock solution prior to SAR-triggering primary infection of *Pma*DG6 (OD_{600}=0.01) or 10mM $MgSO_4$. Then distal leaves (2°, the 6th-8th leaves) without DEX treatment were inoculated with *Pma*DG3 (OD_{600}=0.0002) for the secondary infection. Photos were taken at 3 days after distal leaves infected by *Pma*DG3 in SAR experiment triggered by *Pma*DG6. The SAR result is representative of 5 independent experiments with similar results. Scale bar, 1 cm.

Supplementary Figure S5

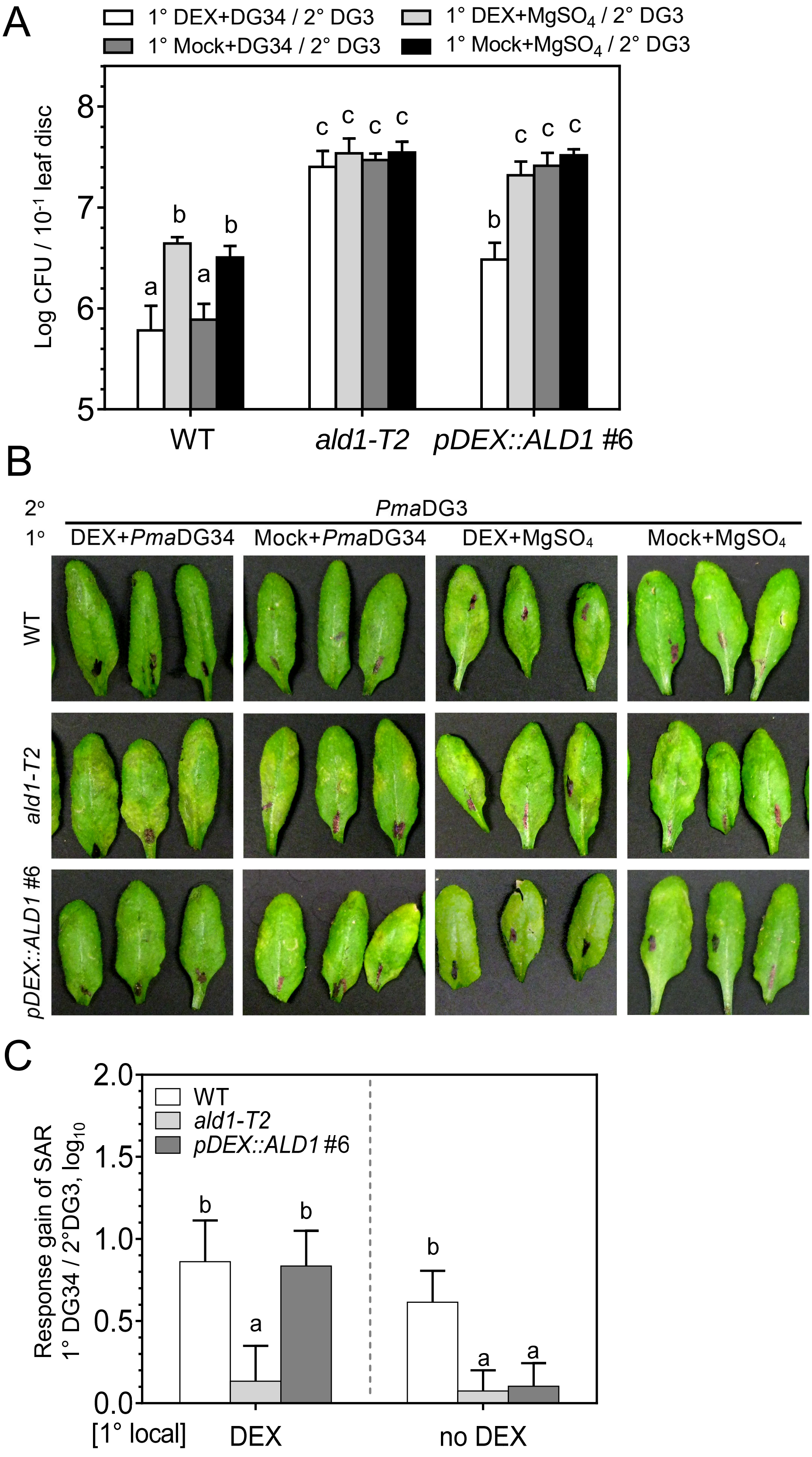

Fig. S5. Restoration of SAR in distal leaves when *Pma*DG34 is used as a primary immunizing infection of DEX-treated local leaves.

(A) SAR response in distal leaves after different treatment in indicated genotypes. Local leaves (1°, the 3rd-5th leaves) were painted with DEX (30 μM) or 10mM $MgSO_4$ for 1 day, and then inoculated with an avirulent strain *Pma*DG34 (DG34, OD_{600}=0.01). Colony-forming unit (CFU) number was measured in distal leaves (2°, the 6th-8th leaves) after secondary infection of *Pma*DG3 (DG3, OD_{600}=0.0002). Error bars indicate SEM of 8 biological replicates (from 8 plants).
(B) Symptoms of representative distal leaves infected with *Pma*DG3 in SAR triggered by *Pma*DG34. Photos were taken about 3 days post infection.
(C) Response gain of SAR in distal leaves infected with *Pma*DG3 (2° DG3) due to 1° infection by *Pma*G34 (1° DG34).
Different letters indicate statistically significant differences (P<0.05, ANOVA, Fisher's LSD test). The result is representative of two independent experiments with similar results.

Supplementary Figure S6

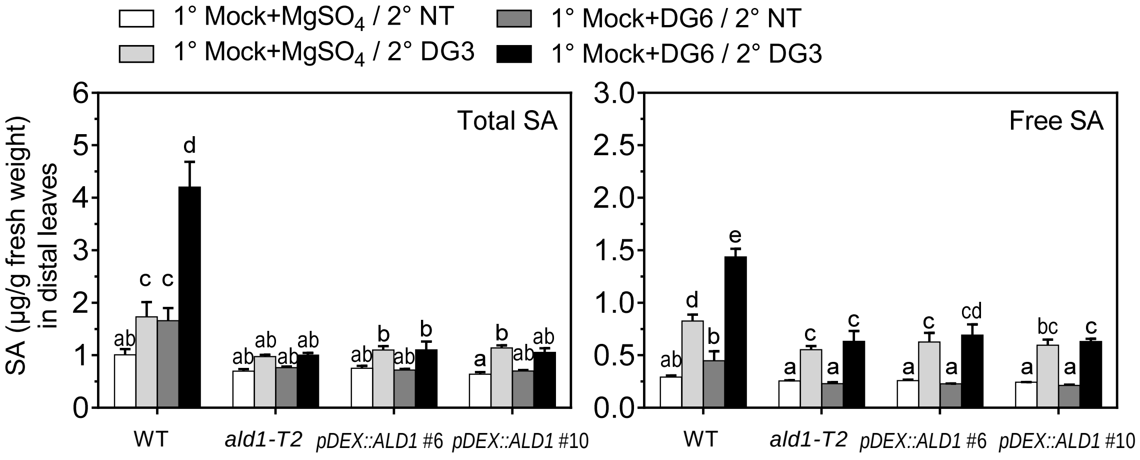

Fig. S6. Lack of restoration of SA accumulation in distal leaves during SAR without DEX treatments of *pDEX::ALD1* lines.

Treatment scheme was as shown in Fig. 6A. Local leaves were treated with mock and then infection by *Pma*DG6 (DG6, OD_{600}=0.01) or 10 mM $MgSO_4$. Then after 1° immunization infection at local leaves for 2 days, distal leaves (without DEX treatment) were then collected at 0 h (NT), or 9 h after 2° infection with *Pma*DG3 (DG3, OD_{600}=0.01). SA levels were measured by HPLC in different genotypes after treatments. Error bars indicate SEM from four biological replicates. Different letters indicate statistically significant differences (P<0.05, ANOVA, Fisher's LSD test). Each biological replicate consists of 6-9 leaves from three plants.

Supplementary Figure S7

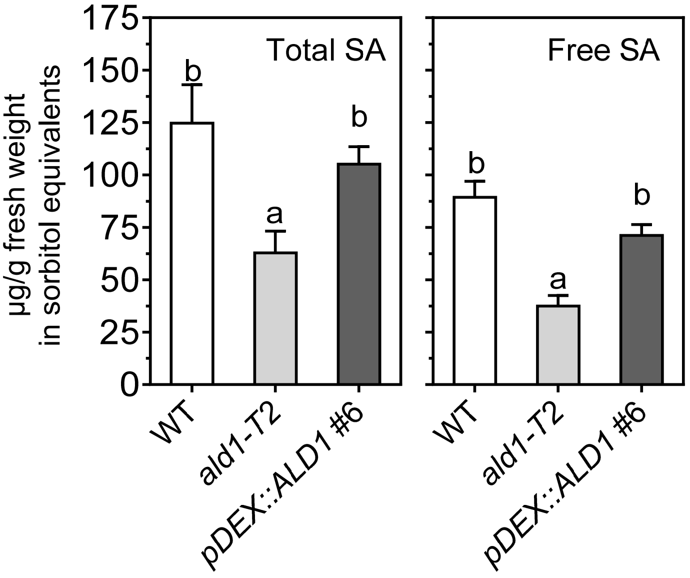

Fig. S7. GC-MS measurements confirms restoration of distal leaf accumulation of SA during SAR activation in *pDEX::ALD1* plants treated with DEX.

After 1° DEX (30 µM) painting for 1 day, local leaves were infection by *Pma*DG6 (OD_{600}=0.01) for 2 days. The distal leaves (without DEX treatment) were then collected at 24 h after 2° infection with *Pma*DG3 (OD_{600}=0.01). Error bars indicate SEM from three biological replicates. Each biological replicate consists of 6-9 leaves from three plants. Different letters indicate statistically significant differences ($P<0.05$, ANOVA, Fisher's LSD test).

Supplementary Table S1. Primer sequences

(1) Primer sequences used for qPCR

Gene Name	Forward primer (5’ to 3’)	Reverse primer (5’ to 3’)
ACTIN2 (At3g18780)	GGTAACATTGTGCTCAGTGGTGG	AACGACCTTAATCTTCATGCTGC
EF1A (At1g07940)	AGGTCCACCAACCTTGACTG	GAGACTCGTGGTGCATCTCA
ALD1 (At2g13810)	TCCCTGATCTGGCTATGACC	GAAACTTCAATCGCGACCTC
PR1 (At2g14610)	TTCTTCCCTCGAAAGCTCAA	AAGGCCCACCAGAGTGTATG
FMO1 (At1g19250)	CCAAACCATTCTTTCGAGGA	CTCAAGCCAAGTTCGGAAAG

(2) Primers for *ALD1* qPCR detection in Figure 1

**Primer name**	**Sequence 5’ to 3’**
ALD1-1F	TCCCTGATCTGGCTATGACC
ALD1-1R	GAAACTTCAATCGCGACCTC
ALD1-2F	TGAGGATTAGTGGGTTTGGACG
ALD1-2R	AGACGATGCACATAACACGAGA
3R-GFPtag-rv	ACAGCTCCTCGCCCTTGCTCA

(3) Primers for *ald1-T2* mutant determination

Forward primer (5’~3’): TTACGATGCATTTGCTATGACC

Reverse primer (5’~3’): TTTTAAATGGAACGCAAGGAG
